# Development of Pseudoginsenoside RT2 as a Novel Gut-Selective Agent: Integrated Pharmacodynamic and Pharmacokinetic Evaluation of an Ocotillol Ginsenoside for Ulcerative Colitis

**DOI:** 10.3390/ph19040622

**Published:** 2026-04-15

**Authors:** Zhuoqiao Li, Junzhe Wu, Jia Wang, Yuwei Liu, Linxuan Liu, Yiyuan Wang, Yanbo Bu, Xiaoyu Geng, Jinping Liu

**Affiliations:** 1 School of Pharmaceutical Sciences, Jilin University, Changchun 130021, China; lizq21@mails.jlu.edu.cn (Z.L.); wujz24@mails.jlu.edu.cn (J.W.); wangjia24@mails.jlu.edu.cn (J.W.); yuwei24@mails.jlu.edu.cn (Y.L.); llx24@mails.jlu.edu.cn (L.L.); yiyuan25@mails.jlu.edu.cn (Y.W.); ybbu25@mails.jlu.edu.cn (Y.B.); 2 School of Pharmacy and Medicine, Tonghua Normal University, Tonghua 134002, China

**Keywords:** ulcerative colitis, Pseudoginsenoside RT2, intestinal barrier, Th17/Treg balance, pharmacokinetics, gut-selective

## Abstract

**Background/Objectives**: Ulcerative colitis is a chronic inflammatory bowel disease marked by a disrupted intestinal barrier and consequent aberrant immune responses. Pseudoginsenoside RT2, an ocotillol-type ginsenoside abundant in Panax herbs, represents a potential therapeutic candidate, yet its anti-ulcerative colitis efficacy and pharmacokinetic profile remain unclear. This study aimed to elucidate RT2’s therapeutic potential for ulcerative colitis through a parallel evaluation of pharmacodynamic efficacy and pharmacokinetic properties. **Methods**: The anti-ulcerative colitis efficacy and in vivo disposition of RT2 were investigated in a trinitrobenzene sulfonic acid-induced rat colitis model. An ultra-performance liquid chromatography–tandem mass spectrometry method was employed to delineate its pharmacokinetic characteristics and quantify its distribution in various tissues following oral administration. **Results**: Pharmacodynamically, RT2 demonstrated significant efficacy in the UC rat model by repairing the intestinal barrier (by promoting goblet cell regeneration and upregulating tight junction proteins and mucin) and restoring immune homeostasis (by correcting T-helper 17/regulatory T-cell imbalance and reducing pro-inflammatory cytokines while elevating anti-inflammatory cytokines). Pharmacokinetically, RT2 exhibited rapid absorption, slow elimination, and high colonic accumulation, with concentrations in the inflamed colon being significantly higher than those in healthy rats. Furthermore, the biphasic concentration–time profile may account for its prolonged systemic residence time and enhanced local exposure. In summary, through parallel efficacy and pharmacokinetic studies, this work systematically reveals its characteristics as a therapeutic agent that exhibits high colonic accumulation and acts via barrier repair and immunomodulation. **Conclusions**: These findings provide a theoretical foundation for the development of RT2 as a novel gut-selective drug candidate for UC.

## 1. Introduction

Ulcerative colitis (UC) is a chronic inflammatory bowel disease marked by continuous mucosal inflammation and ulceration, leading to symptoms such as abdominal pain, diarrhea, and bloody stools [1,2]. Pathogenesis involves genetic, microbial, immune, and environmental factors, with a breached intestinal barrier triggering aberrant immunity as a central mechanism [3,4]. Current pharmacological therapy for UC includes aminosalicylates, glucocorticoids, immunosuppressants, and biologics [5]. However, there remain unmet clinical needs; for instance, some patients respond inadequately to existing treatments or lose response over time, and clinical remission does not equate to barrier protection, which is a critical goal for reducing the risk of recurrence and carcinogenesis. Consequently, the focus of drug development has shifted from clinical remission to barrier protection. Intestinally selective drugs that achieve colon-enriched distribution and enhance barrier function have thus become a key priority in research and development. The strategy for drug discovery has also shifted from a sole focus on activity to a balanced emphasis on both activity and pharmacokinetic properties.

Pharmacokinetic (PK) studies are a fundamental component of drug development, as they allow for characterizing the absorption, distribution, metabolism, and excretion (ADME) of a compound, as well as establishing dose–response and concentration–time relationships [6,7]. Among these, the study of drug absorption and distribution is a critical step in elucidating drug efficacy and safety. Absorption research focuses on the dynamic process by which a drug enters the systemic circulation from its administration site. Its core lies in analyzing the rate and extent of this process, which is ultimately quantified through key parameters such as bioavailability, thereby revealing the initial efficiency and completeness of the drug’s therapeutic action. Distribution research, on the other hand, aims to characterize the spatial dispersion and equilibrium state of the drug within the body. By examining its transport and accumulation patterns across different tissues and body fluids, it clarifies whether effective exposure can be achieved at the target organs and provides early warnings of potential safety risks associated with off-target accumulation. Together, these two areas of research form the foundational characteristics of a drug’s in vivo behavior, providing core evidence for efficacy prediction and risk assessment.

Ocotillol-type ginsenosides possess higher lipid solubility than dammarane-type ginsenosides, promoting membrane penetration, accelerating absorption, slowing elimination, and ultimately enhancing oral bioavailability and bioactivity [8,9]. Representative compounds such as pseudoginsenoside RT4, pseudoginsenoside F11, and majoroside R2 exhibit broad pharmacological properties, including anti-inflammatory, intestinal barrier protection, neuroprotection, and cardioprotective effects [10,11,12,13]. In particular, RT4 has been shown to alleviate UC progression by inhibiting inflammatory responses, reinforcing the intestinal barrier, and restoring gut microbiota balance [14]. Pseudoginsenoside RT2 (RT2) is a rare ocotillol-type ginsenoside present in several Panax species, such as Panax pseudoginseng subsp. Himalaicus, Panax quinquefolius L., Panax notoginseng, and Panax japonicus var. major [15,16,17]. It is also present at lower levels in steamed ginseng and steamed American ginseng berry [18]. Its natural abundance is low, resulting in limited pharmacological characterization. Currently, only one study has reported its antiplatelet activity in vitro. In platelet aggregation assays induced by adenosine diphosphate (ADP) and arachidonic acid (AA), RT2 exhibited moderate inhibitory activity against ADP-induced aggregation, whereas its effect on AA-induced aggregation was negligible; the IC_50_ values of RT2 were determined to be 90.35 ± 1.05 μmol/L and >100 μmol/L, respectively [16]. However, whether RT2 exerts a direct protective effect in the UC model, whether its mechanism involves the modulation of the intestinal barrier, and whether it has good pharmacokinetic properties and can achieve enriched distribution in the colon remain to be elucidated.

In this study, the anti-inflammatory and barrier-protective effects of RT2 were evaluated in a 2,4,6-trinitrobenzene sulfonic acid (TNBS)-induced UC rat model, which closely mirrors the Th17-driven immunopathology seen in human UC. The disease activity index (DAI), colon length, chronic disease management index (CMDI), spleen coefficient, inflammatory and oxidative stress indicators, histopathological changes, ultrastructural integrity of intestinal epithelial cells, tight junction protein (TJ) and mucin expression, number and proportion of Th17 and Treg cells were assessed to evaluate the therapeutic effects of RT2 on TNBS-induced colitis. To evaluate colon-enriched efficiency, the distribution of RT2 in the colon was quantified using pharmacokinetic analysis after oral administration. Furthermore, the absorption and distribution of RT2 were characterized. Dose–response and concentration–time relationships were also established. Collectively, these pharmacodynamic and pharmacokinetic results provide the first evidence supporting RT2 as a promising therapeutic candidate for UC, with a colon-enriched distribution and enhanced barrier-protective functions.

## 2. Results

### 2.1. RT2 Alleviated TNBS-Induced UC in Rats

#### 2.1.1. Effects on Clinical Parameters and Colonic Injury

**Figure 1:**
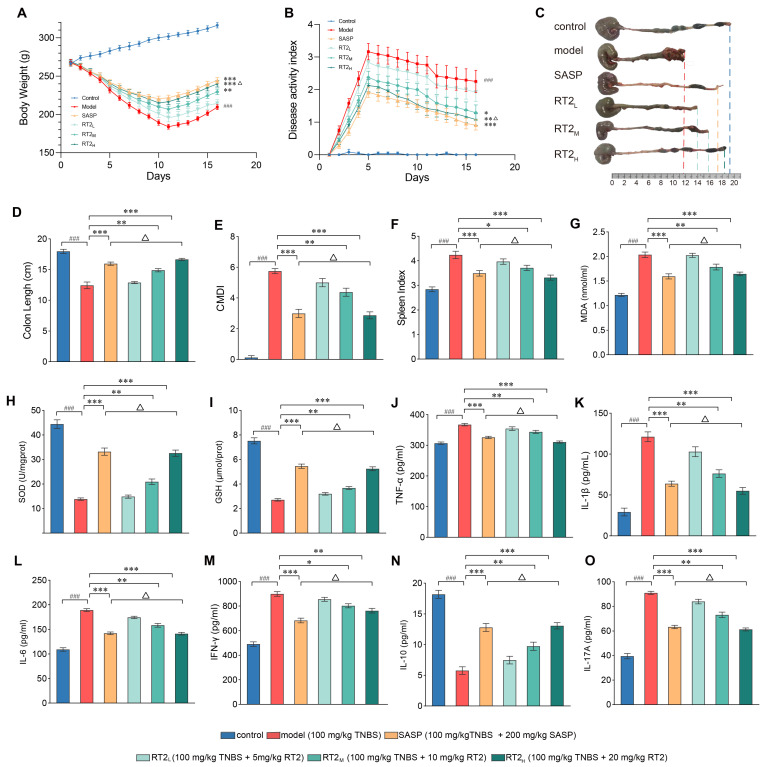
Effects of RT2 on clinical parameters, colonic injury, oxidative stress, and inflammation in TNBS-induced UC rats. (**A**) Body weight changes; (**B**) DAI scores; (**C**) representative colonic images and (**D**) colon length measurements; (**E**) CMDI scores; (**F**) the spleen index; (**G**–**I**) colonic levels of oxidative stress indicators: MDA, SOD, and GSH; (**J**–**O**) levels of inflammatory cytokines (TNF-α, IL-1β, IL-6, IFN-γ, IL-10, and IL-17A) in colon tissue. Data are presented as mean ± SEM. ^###^ *p* < 0.001 versus control group; * *p* < 0.05, ** *p* < 0.01, *** *p* < 0.001 versus model group; Δ *p* > 0.05 versus SASP group.

As shown in Figure 1A,B, control rats exhibited steady weight gain and consistently low DAI scores, with no signs of diarrhea or bleeding. In contrast, TNBS administration induced characteristic UC symptoms, including significant weight loss, diarrhea and hematochezia, leading to significantly elevated DAI scores in the model group (*p* < 0.001). Compared with the model group, both sulfasalazine (SASP) and RT2 treatments significantly attenuated weight loss and reduced DAI scores (*p* < 0.05 to *p* < 0.001). RT2 demonstrated a dose-dependent effect, with the high-dose RT2 (RT2_H_) group showing efficacy comparable to that of the SASP group (*p* > 0.05). These results indicate that RT2 effectively alleviated UC-related symptoms in a dose-dependent manner.

Colon length was significantly shortened in TNBS-induced rats compared with controls (*p* < 0.001), indicating severe colonic damage. Treatment with either SASP or RT2 significantly restored colon length (*p* < 0.01 and *p* < 0.001, respectively), with the RT2_H_ group reaching a length similar to that of the SASP group (Figure 1C,D).

Histological examination revealed an intact colonic mucosa in control animals, without evidence of erosion, ulceration, congestion, or edema. The model group, however, displayed severe mucosal damage, including extensive congestion, edema, surface necrosis, and multiple ulcerations. RT2 treatment markedly ameliorated these pathological changes, reducing congestion and edema, promoting tissue repair, and resulting in only mild focal erosions or occasional small ulcers. The chronic disease management index (CDMI) score is shown in Figure 1E.

The spleen index, an indicator of systemic inflammation, was significantly higher in the model group than in the control group (*p* < 0.001). Both SASP and RT2 treatments significantly reduced this increase (*p* < 0.05 and *p* < 0.001, respectively), with RT2_H_ showing a restorative effect similar to that of SASP (*p* > 0.05; Figure 1F).

Body weight and DAI scores can be used to assess the daily condition of rats. The colon is the primary lesion site in UC, where inflammatory edema, smooth muscle spasms and contractions, and epithelial damage can lead to shortening of the colon length. Therefore, colon length serves as the most intuitive macroscopic indicator of disease severity. The spleen index reflects the level of systemic inflammation and immune function. Detection of these indicators demonstrated that RT2 can reverse the TNBS-induced reduction in body weight, elevation of DAI scores, decrease in colon length, and increase in spleen coefficient, thereby alleviating UC clinical symptoms and colonic damage.

#### 2.1.2. Effects on Inflammatory Cytokines and Oxidative Stress Marker Levels

TNBS administration significantly altered the profile of inflammatory cytokines in colon tissue compared with the control group. In colon tissue, RT2 and SASP also effectively modulated oxidative stress parameters. Both treatments significantly increased the activities of the antioxidant enzymes glutathione peroxidase (GSH) and superoxide dismutase (SOD) while reducing the level of the lipid peroxidation product malondialdehyde (MDA) (*p* < 0.05 to *p* < 0.001). The high-dose RT2 group (RT2_H_) exhibited effects comparable to those of the SASP group (Figure 1G–I).

TNBS administration significantly altered the profile of inflammatory cytokines in colon tissue compared with the control group. Levels of the anti-inflammatory cytokine IL-10 were markedly reduced, while those of pro-inflammatory cytokines (interleukin-1β (IL-1β), IL-6, IL-17A, tumor necrosis factor-α (TNF-α), and interferon-γ (IFN-γ)) were substantially elevated (*p* < 0.001). Treatment with SASP or RT2 significantly reversed these changes in a dose-dependent manner (*p* < 0.05 to *p* < 0.001) (Figure 1J–O).

In summary, RT2 treatment rebalanced inflammatory cytokine responses and alleviated oxidative stress in TNBS-induced UC, demonstrating protective effects comparable to those of the reference drug SASP.

#### 2.1.3. Effects on Colonic Histopathology

**Figure 2:**
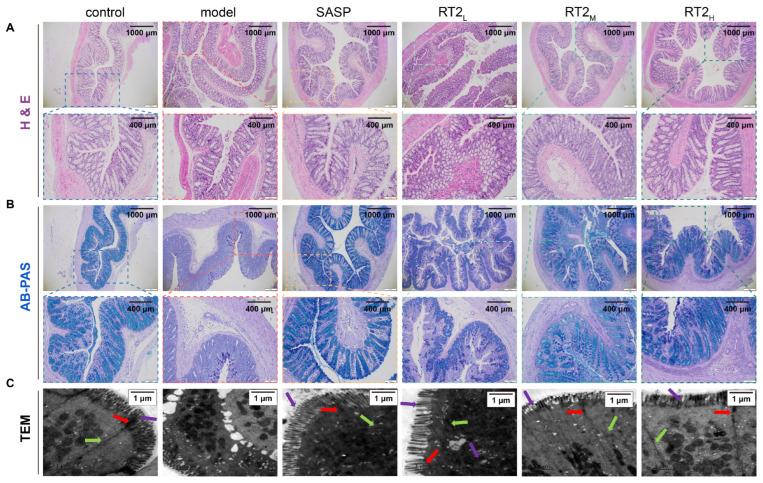
Histopathological and ultrastructural evaluation of colonic tissue. Representative micrographs of colon sections from each group: (**A**) H&E staining; (**B**) AB-PAS staining; (**C**) transmission electron microscopy (TEM) images of the colonic epithelium. Different colored arrows indicated the following structures: purple arrows, microvilli; red arrows, intact tight and green arrows, gap junctions. Scale bars are indicated in each panel.

Histopathological changes were assessed by hematoxylin and eosin (H&E) staining, Alcian Blue–Periodic Acid–Schiff (AB-PAS) staining, and transmission electron microscopy (TEM). Colon sections from control rats exhibited a normal mucosal architecture, characterized by an intact epithelial lining, well-ordered crypt glands, and abundant goblet cells (Figure 2A). In contrast, TNBS-induced model rats showed severe mucosal damage, including a distorted crypt architecture, crypt abscesses, submucosal edema, dense inflammatory cell infiltration, and a marked depletion of goblet cells. Treatment with either SASP or RT2 significantly ameliorated these histopathological alterations. The therapy groups showed notable mucosal repair, reduced inflammatory infiltration and edema, and a partial restoration of goblet cell numbers, indicating effective attenuation of TNBS-induced colonic injury.

AB-PAS staining, which specifically highlights mucin-producing goblet cells, further corroborated these findings (Figure 2B). Control rats presented with regularly arranged, mucin-rich goblet cells within intact crypts. The model group, however, showed disrupted crypt morphology and a marked reduction in both goblet cell numbers and intracellular mucin content, accompanied by edema and inflammation. Compared to the model group, colon sections from SASP- and RT2-treated rats exhibited a restored crypt structure and a marked increase in goblet cell abundance and mucin production, indicating improved mucosal barrier protection.

TEM revealed regular microvilli (purple arrows), intact tight (red arrows) and gap junctions (green arrows), and mitochondria with well-defined cristae in the colonic epithelium of control rats. In contrast, the model group exhibited sparse/shortened microvilli, diminished intercellular junctions, reduced and vacuolated mitochondria, and heterogeneous cytoplasm. Treatment with RT2 or SASP markedly restored these ultrastructural features, improving microvilli organization and junctional integrity, with RT2 demonstrating a dose-dependent effect (Figure 2C). These findings further support the protective effect of RT2 on intestinal mucosal barrier integrity.

#### 2.1.4. Effects on the Expression of Tjs and Mucin

**Figure 3:**
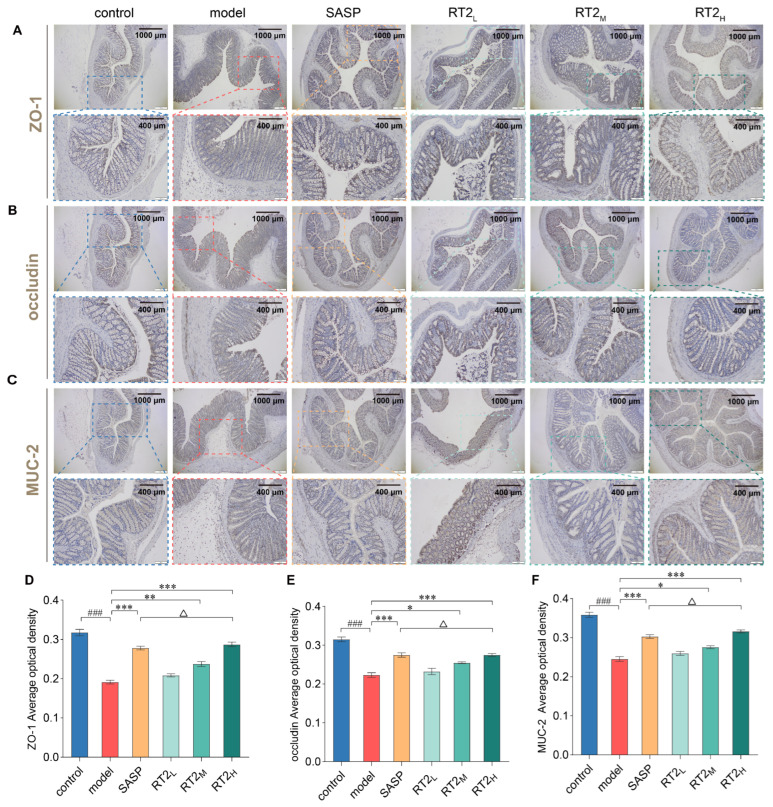
Representative immunohistochemical staining of (**A,D**) ZO-1, (**B,E**) Occludin and (**C,F**) MUC2. Data are presented as mean ± SEM. ^###^ *p* < 0.001 versus control group; * *p* < 0.05, ** *p* < 0.01, *** *p* < 0.001 versus model group; Δ *p* > 0.05 versus SASP group.

Immunohistochemical staining was performed to evaluate the expression and localization of key TJs (ZO-1, Occludin, Claudin-1, E-cadherin) and the mucin MUC2. As shown in Figure 3A–F and Figure S6, immunohistochemical staining for TJ and mucin (ZO-1, Occludin, Claudin-1, E-cadherin, MUC2) control samples exhibited strong and continuous immunoreactivity for all markers, indicating intact mucosal integrity. In stark contrast, the model group showed markedly attenuated and discontinuous staining patterns, consistent with severe impairment of the intestinal barrier. Treatment with either SASP or RT2 effectively restored the intensity and continuity of the staining for these proteins. Quantitative analysis further confirmed these observations. The expression levels of all five proteins were significantly downregulated in the model group compared to the controls (*p* < 0.001). This downregulation was significantly reversed following intervention with SASP or RT2. Notably, the high-dose RT2 (RT2_H_) group restored protein expression to levels comparable to, or even exceeding, those observed in the SASP-treated group. These results demonstrate that RT2 treatment, particularly at a higher dose, effectively promotes the repair of the intestinal barrier in this rat model of UC.

#### 2.1.5. Effects on Th17 and Treg Cell Populations

**Figure 4:**
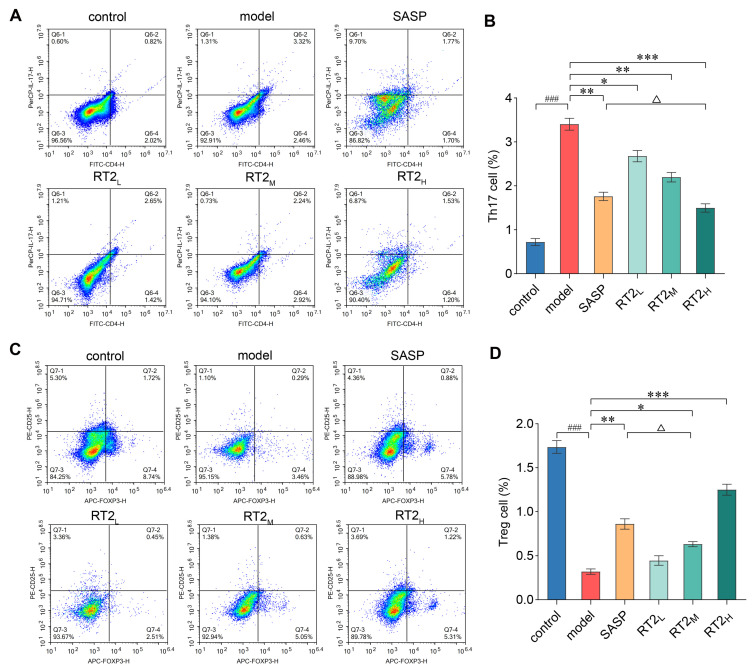
Flow cytometric quantification of Th17 and Treg cell populations. (**A**,**B**) Representative plots and summary data for CD4^+^ IL-17A^+^ Th17 cells. (**C**,**D**) Representative plots and summary data for CD4^+^ CD25^+^ Foxp3^+^ Treg cells. Data are presented as mean ± SEM. ^###^ *p* < 0.001 versus control group; * *p* < 0.05, ** *p* < 0.01, *** *p* < 0.001 versus model group; Δ *p* > 0.05 versus SASP group.

Given the central role of Th17/Treg immune dysregulation in UC, we quantified these lymphocyte subsets in mesenteric lymph nodes (mLNs) by flow cytometry. Relative to the controls, the model group exhibited a significantly increased Th17 cell proportion and a concurrent reduction in Treg cells (*p* < 0.001). Treatment with either SASP or RT2 effectively reversed this imbalance in a dose-dependent manner (Figure 4). The observed cellular shifts aligned with the cytokine alterations noted in Section 2.1.2, wherein RT2 administration reduced pro-inflammatory cytokines linked to Th17 cells and increased anti-inflammatory cytokines associated with Treg cells. Collectively, these findings demonstrate that the therapeutic effect of RT2 in TNBS-induced UC is mediated, at least in part, through the restoration of the Th17/Treg equilibrium.

#### 2.1.6. Tissue Distribution of RT2 in UC Rats

**Figure 5:**
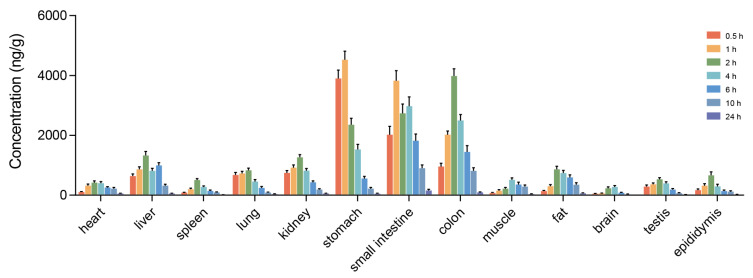
Tissue distribution of RT2 in UC rats.

To elucidate the in vivo disposition of RT2, its concentrations in various tissues were quantified following a single intragastric administration (20.0 mg/kg) to TNBS-induced UC rats. As shown in Figure 5, RT2 reached peak levels in most tissues within 1–2 h post-administration. The compound exhibited a pronounced distribution to the gastrointestinal tract. It was primarily detected in the stomach, small intestine, and colon during the first 0.5–1 h, with a subsequent shift showing predominant accumulation in the colon by 2 h. The highest concentration was observed in the small intestine and colon within 4 to 24 h post-administration. Meanwhile, a transient rise in RT2 levels was detected in the liver and small intestine at 6 h, potentially attributable to the enterohepatic circulation. Notably, RT2 demonstrated a higher affinity for inflamed colonic tissue, suggesting a targeted accumulation at the primary site of pathology, which may underpin its rapid local pharmacological action.

### 2.2. Pharmacokinetic Evaluation of RT2: In Vivo Profiling and Assessment of Druggability

To comprehensively characterize the in vivo pharmacokinetic profile and assess the druggability of RT2, a detailed pharmacokinetic study was performed. The concentrations of RT2 and its major metabolites (RT5 and ocotillol) were quantified in biological samples (plasma and tissues) collected from normal rats using a validated UPLC-MS/MS method.

#### 2.2.1. Validation of the Liquid Chromatography–Tandem Mass Spectrometry (LC-MS/MS) Analytical Method

Specificity: Multiple reaction monitoring (MRM) is a highly sensitive and specific quantitative detection mode based on triple quadrupole mass spectrometry. Unlike conventional UV detection, which relies solely on chromatographic separation, MRM achieves compound identification through two stages of mass filtering: selection of precursor ions in Q1 and monitoring of characteristic product ions in Q3 after collision-induced dissociation. This dual-filtering mechanism provides exceptional specificity and anti-interference capability, even when chromatographic separation is incomplete [19,20]. Under the optimized chromatographic conditions, RT2, RT5, ocotillol, and the internal standard (IS) (Re) exhibited sharp peaks with retention times of 2.14, 2.22, 3.21, and 1.58 min, respectively. Representative chromatograms of blank matrices (plasma, liver, and colon), blank matrices spiked with the analytes and IS, and actual study samples collected at 2 h or 4–8 h post-oral administration of RT2 are shown in Figure S8. No significant endogenous interference was observed at the retention times corresponding to the analytes or the IS in any of the tested matrices. The baseline remained stable across all chromatograms. These results confirm the high specificity of the developed method for the reliable quantification of RT2 and its metabolites in complex biological samples.

Calibration curve, linearity, and lower limit of quantification (LLOQ): Calibration curves for RT2, RT5, and ocotillol were successfully established in all investigated matrices (plasma and 13 tissues). Each analyte demonstrated good linearity over a broad concentration range of 5–5000 ng/mL. The corresponding regression equations, correlation coefficients (R^2^), and specific linear ranges for each matrix are comprehensively presented in Table S2. The LLOQ was set at 5 ng/mL for all analytes. Method validation at this concentration confirmed the reliability of the assay for low-level detection. The accuracy at the LLOQ fell within the accepted criterion of 80–120% of the nominal value, while the precision, expressed as RSD, was below 20%. These results confirm the sensitivity and reliability of the method for accurate quantification across the intended dynamic range.

Precision and Accuracy: The intra-day and inter-day precision and accuracy were assessed using quality control (QC) samples. As presented in Table S3, the precision (RSD) for all analytes (RT2, RT5, and ocotillol) across different matrices (plasma, liver, and colon homogenates) was below 15%, and the accuracy ranged from 85% to 115%, demonstrating the reliability of the method.

Dilution reliability: The integrity sample dilution was assessed by evaluating a 5-fold dilution scheme. Plasma samples spiked at a concentration of 7500 ng/mL were diluted with blank plasma to achieve a final concentration of 1500 ng/mL. As presented in Table S4, the precision (expressed as RSD) was below 15%, and accuracy ranged within 85–115% for all analytes (RT2, RT5, and ocotillol). These results confirm that dilution did not compromise the accurate quantification of the analytes in plasma.

Extraction recovery and matrix effect: The extraction recovery and matrix effect were evaluated across all biological matrices (plasma, liver, and colon tissue homogenates). As summarized in Table S5, the mean extraction recoveries for RT2, RT5, and ocotillol ranged from 86.21% to 104.65%, with RSD values below 15%. The matrix effect, expressed as the matrix factor, fell within 86.87% to 111.64% (RSD < 15%), indicating negligible interference from the matrices on analyte ionization. These results demonstrate that the sample preparation procedure is efficient and that the quantification of the analytes is not adversely affected by matrix components.

Stability: The stability of RT2, RT5, and ocotillol was comprehensively evaluated under a range of conditions relevant to sample handling and storage. This included short-term (bench-top) stability at room temperature, long-term stability at −80 °C, stability after multiple freeze–thaw cycles, post-preparation stability in the autosampler, and stock solution stability. As shown in Table S6, for both low and high QC levels, all analytes demonstrated acceptable stability. The measured accuracy remained within 85%~115%, and precision (RSD) was below 15% across all tested conditions, confirming the reliability of the analytical method throughout the sample lifecycle.

Residual effect: Carryover was assessed by analyzing blank matrix samples (plasma, liver, and colon tissue homogenates) immediately following three consecutive injections of the ULOQ standard. No significant interfering peaks were observed at the retention times corresponding to the analytes (RT2, RT5, and ocotillol) or the internal standard in any blank matrix. The response in blank samples was less than 20% of the LLOQ response for the analytes and less than 5% for the IS, meeting the pre-defined acceptance criteria. This confirms the absence of significant carryover that could impact sample quantification.

#### 2.2.2. Absorption Study of RT2

**Figure 6:**
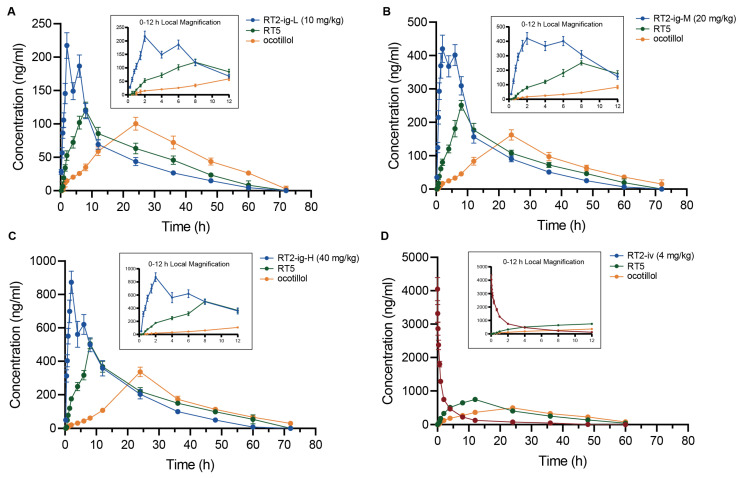
Mean plasma concentration–time curves of RT2, RT5, and ocotillol. (**A**–**C**) Curves receiving oral doses of 10, 20, and 40 mg/kg; (**D**) Curve receiving an intravenous dose of 4 mg/kg.

The plasma concentration–time profiles of RT2 and its metabolites (RT5 and ocotillol) following oral and intravenous administration are presented in Figure 6. The pharmacokinetic parameters of RT2, calculated using a compartmental model in DAS 2.0 software, are summarized in Table 1.

After oral administration of RT2 at doses of 10, 20, and 40 mg/kg, the peak plasma concentrations were achieved rapidly, with Tmax values ranging from 1.83 to 2.00 h. The area under the curve AUC_0–∞_ values for the low- (10 mg/kg), medium- (20 mg/kg), and high-dose (40 mg/kg) groups were 3207.37 ± 141.64, 6929.95 ± 576.26, and 13,310.78 ± 1233.36 μg·h/L, respectively. Dose-normalized AUC (AUC/dose) values were 320.74 ± 14.16, 346.50 ± 28.81, and 332.77 ± 30.83 μg·h/L per mg/kg, showing no significant differences among the three groups (*p* > 0.05). Similarly, dose-normalized C_max_ remained consistent across doses (*p* > 0.05). These data indicate that RT2 exhibited linear pharmacokinetics within the dose range of 10–40 mg/kg, consistent with the compartmental modeling results. The elimination was relatively slow, with half-lives (t_1/2_) between 12.30 and 13.61 h, and mean residence times (MRT_0-t_) from 14.22 to 15.30 h. The apparent volume of distribution (V_d_: 52.91–61.51 L/kg) and clearance (CL: 2.90–3.12 L/h/kg) suggest an extensive tissue distribution, consistent with the compound’s lipophilic properties. Consistent with the dose-proportional exposure, the compartmental analysis revealed no significant dose-dependent changes in clearance or elimination half-life. A biphasic profile was observed in the concentration–time curve following oral dosing, characterized by an initial peak at 1.5–2 h and a distinct secondary peak at around 6 h post-dose. This pattern may be indicative of enterohepatic recirculation, a process that could potentially prolong systemic exposure and contribute to sustained plasma concentrations. However, direct experimental confirmation is required. Analysis of the metabolites RT5 and ocotillol revealed sequential pharmacokinetics. RT5 reached its peak concentration (T_max_) at approximately 8 h post-dose, while ocotillol peaked at around 24 h. This indicates a dynamic shift in the circulating species over time: RT2 predominates initially, RT5 becomes the major component between 12 and 24 h, and ocotillol prevails thereafter. This sequential transition indicates a time-dependent shift in the predominant circulating species from RT2 to its metabolites RT5 and ocotillol. Whether these metabolites contribute to the overall pharmacological effects of RT2 remains to be investigated in future studies.

Following intravenous administration (4 mg/kg), the plasma AUC_0–t_ of RT2 was 8782.73 ± 436.16 μg·h/L. The absolute oral bioavailability (F) was calculated to be 14.45 ± 0.65%. While the bioavailability of ginsenosides is typically low due to their high molecular weight, extensive glycosylation, and pre-systemic metabolism by gut microbiota, the moderately higher lipophilicity of RT2 may contribute to its comparatively enhanced intestinal permeability and bioavailability relative to other ginsenosides.

#### 2.2.3. Tissue Distribution of RT2

**Figure 7:**
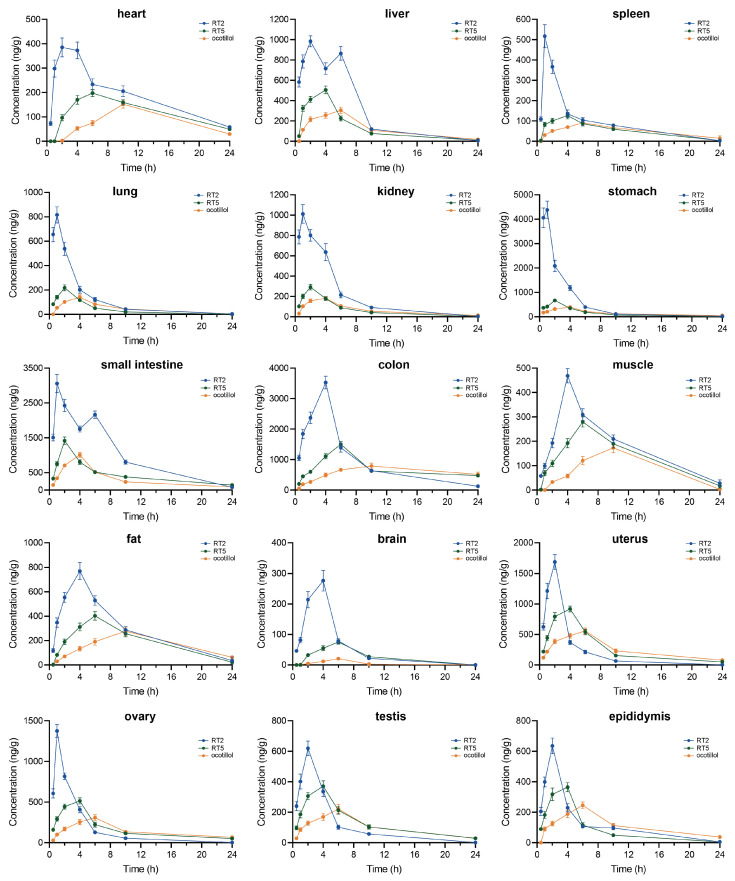
Tissue distribution profiles of RT2, RT5, and ocotillol in rats following a single oral administration of RT2 (20 mg/kg). Data points represent mean concentrations (*n* = 6) at each time point in the indicated tissues.

The distribution profiles of RT2 and its metabolites (RT5 and ocotillol) were investigated in 15 rat tissues (including the heart, liver, spleen, lung, kidney, stomach, small intestine, colon, muscle, fat, brain, uterus, ovary, testis, and epididymis) at seven time points following a single oral dose of 20 mg/kg (Figure 7). RT2 was rapidly and extensively distributed to all tissues examined.

The concentration–time trends in most tissues mirrored the plasma profile, with peak concentrations occurring between 1 and 4 h post-dose, suggesting that the systemic circulation primarily governs the initial tissue distribution. However, distinct tissue-specific patterns were observed. At early time points (0.5–2 h), RT2 predominantly accumulated in the gastrointestinal tract (stomach, small intestine, and colon). Between 2 and 6 h, the highest concentrations were detected in the small intestine and colon, with levels in the colon exceeding those in the small intestine. This pronounced localization to gastrointestinal tissues aligns with the large apparent volume of distribution estimated in the pharmacokinetic study and may facilitate local pharmacological action.

A kinetic profile consistent with the enterohepatic circulation was observed in the liver and small intestine; however, direct experimental confirmation is needed to support this interpretation. The hepatic concentration of RT2 peaked at 2 h, declined, and then showed a secondary increase at 6 h, consistent with re-entry via the portal circulation following intestinal reabsorption. Correspondingly, the small intestine exhibited a biphasic concentration profile: an initial peak at 1 h (from the unabsorbed drug and initial biliary excretion) and a subsequent peak resulting from continuous biliary excretion and subsequent reabsorption.

Meanwhile, the metabolites RT5 and ocotillol also demonstrated an extensive tissue distribution, with their concentrations in most tissues exceeding those in plasma, indicating a significant extravascular distribution and tissue sequestration. A key finding was the differential distribution between plasma and tissues. While metabolites (RT5 and ocotillol) became the predominant circulating species in plasma after 12 h, the parent drug RT2 remained the major component in most tissues throughout the observation period. This suggests that RT2 itself is likely the principal pharmacologically active form at the target tissue sites, and its significant and sustained presence in the gastrointestinal tract may underpin localized therapeutic effects.

## 3. Discussion

To comprehensively evaluate the therapeutic potential of RT2, this study employed an integrated experimental strategy in rat models, enabling the concurrent assessment of its in vivo pharmacological efficacy and preliminary pharmacokinetic behavior. Anti-ulcerative colitis activity was investigated in a well-established disease model, while its absorption and distribution profiles were characterized in healthy rats following intragastric administration. This parallel approach provides a more holistic initial understanding of the compound’s bioactivity and systemic exposure. A critical aspect of this work was the selection of an appropriate experimental model for UC. To authentically assess therapeutic potential, we employed the TNBS/ethanol-induced UC model, which recapitulates key clinical features—initial barrier disruption followed by hapten-driven, chronic transmural inflammation. In this model, RT2 demonstrated significant efficacy, ameliorating disease severity (reduced the DAI, CDMI, spleen coefficient, and colon shortening) comparably to sulfasalazine. Pharmacodynamic evaluation revealed that RT2 treatment was associated with a dual pattern of effects. First, it coincided with enhanced barrier integrity, as reflected by increased goblet cell counts, improved mucosal and ultrastructural morphology (including microvilli and intercellular junctions), and upregulated expression of tight junction proteins and mucins. Second, it paralleled a rebalancing of the Th17/Treg immune axis, accompanied by reduced levels of pro-inflammatory cytokines (IL-1β, IL-6, IL-17A, TNF-α, and IFN-γ) and increased expression of the anti-inflammatory cytokine IL-10. These findings provide pharmacodynamic evidence that RT2 acts in concert with improvements in both epithelial barrier function and immune regulation, warranting further mechanistic investigation.

The observed efficacy is strongly supported by a well-characterized and advantageous pharmacokinetic (PK) profile. RT2 exhibits rapid absorption (T_max_, 1.5–2 h), slow elimination (t_1/2_, 12.3–13.6 h), linear dose–exposure profile, and a large volume of distribution (V_d_, 52.9–61.5 L/kg), consistent with the enhanced lipophilicity imparted by its ocotillol core relative to dammarane-type ginsenosides. Critically, tissue distribution studies revealed a pronounced colonotropic tendency. RT2 preferentially accumulated and was retained in the colon over 2–10 h post-dose. Notably, colonic concentrations were significantly higher in UC rats than in healthy controls, suggesting inflammation-enhanced, site-specific retention. Inflammation increased local blood flow and enhanced vascular permeability, and inflammatory cell binding may have contributed to the high distribution of RT2 to the diseased colon. This property may contribute to optimal drug availability at the primary lesion site. The high accumulation of RT2 in the colon is likely attributable to passive factors, including differential tissue perfusion, the presence of inflammatory sites that may enhance drug retention, or potential interactions with gut microbiota, rather than an active targeting mechanism. A biphasic concentration–time profile was observed in plasma, the liver, and the small intestine, which may be suggestive of enterohepatic circulation. One possible interpretation involves a cycle of hepatic conjugation, biliary excretion, intestinal deconjugation (potentially microbiota-mediated), and subsequent reabsorption. Such a process could prolong systemic residence time, increase overall exposure, and potentially sustain colonic drug levels, thereby contributing to the durable pharmacodynamic effects observed. However, direct experimental validation is required to substantiate this hypothesis.

By adopting this parallel pharmacokinetic and pharmacodynamic evaluation, this study establishes a foundational framework for understanding RT2’s in vivo performance. The efficacy data generated in a pathologically relevant model offer meaningful insights into its potential therapeutic utility against UC. Simultaneously, the pharmacokinetic parameters obtained from healthy rats, such as the absorption rate and tissue distribution, provide crucial preliminary data on its systemic behavior, informing future dose selection and mechanistic studies. This combined methodology not only accelerates the preliminary characterization of RT2 but also strengthens the translational relevance of the findings, paving the way for more targeted and in-depth investigations into its mechanism of action and overall drug-like profile.

While this study provides compelling evidence for the therapeutic potential of RT2, several limitations should be acknowledged to properly contextualize the findings and guide future research. The pharmacodynamic validation was conducted exclusively in vivo. Although the integrated rat model offers physiological relevance, the precise molecular mechanisms, including the direct cellular targets and downstream signaling pathways through which RT2 modulates epithelial repair and immune rebalancing, remain to be delineated. Future work should employ in vitro models combined with techniques such as RNA sequencing, proteomics, or target deconvolution strategies to elucidate the exact mechanistic underpinnings of its action. Moreover, the observed biphasic pharmacokinetic profile suggests the involvement of the enterohepatic circulation, a feature that could significantly enhance its sustained local exposure. However, this hypothesis remains inferential. Definitive confirmation requires targeted experimental validation, such as comparative pharmacokinetic studies in bile duct-cannulated versus sham-operated rats or the administration of RT2 in combination with inhibitors of key processes in the EHC loop (e.g., biliary excretion or intestinal reabsorption). Establishing the EHC definitively will clarify its contribution to RT2’s pharmacokinetic and pharmacodynamic profile. Finally, comprehensive safety and contraindication studies, including repeated-dose toxicity, reproductive toxicity, central nervous system safety pharmacology, and drug interaction assessments, are necessary before RT2 can be considered a clinically viable alternative to traditional ginsenosides.

## 4. Materials and Methods

### 4.1. Materials and Animals

RT2 was prepared in our laboratory (its structure was unequivocally confirmed through a comprehensive spectroscopic analysis, including HR-MS, ^1^H-NMR, ^13^C-NMR, HMQC, and HMBC spectroscopy, the charts for which are shown in the Figures S1–S5 and Table S1 in Supplementary Materials. TNBS was purchased from Sigma-Aldrich Company (Shanghai, China). SASPs were purchased from Bide Pharmatech Co., Ltd. (Shanghai, China). A series of enzyme-linked immunosorbent assay (ELISA) kits for the quantification of cytokines and oxidative stress markers, including IL-1β, IL-6, IL-10, IL-17A, IFN-γ, TNF-α, SOD, MDA, and GSH-Px, were purchased from Feiya Biotechnology Co., Ltd. (Nantong, Jiangsu, China). GolgiPlug™, FITC-anti-rat CD4, PE-anti-rat CD25, Alexa Fluor 647-anti-rat IL-17, and PerCP-anti-rat Foxp3 were purchased from Becton, Dickinson and Company (Franklin Lakes, NJ, USA). Methanol and acetonitrile were purchased from Thermo Fisher Scientific (Waltham, MA, USA). Water was purchased from A.S.Waston TM Limited (Hong Kong, China).

Specific pathogen-free (SPF) SD rats (male, 200 ± 20 g) were supplied by Beijing Huafukang Biological Technology Co. Ltd. (Beijing, China, License No.: SCXK(jing)2024-0003). A total of 180 rats were used in this study. The rats were housed in the Barrier Environment Animal Experiment Center under a 12 h light/dark cycle, with the temperature maintained at 25 ± 2 °C and relative humidity at 60 ± 5%. All experimental procedures were reviewed and approved by the Ethics Committee of the Jilin University School of Pharmaceutical Sciences.

### 4.2. Effect of RT2 on TNBS-Induced UC in Rats

#### 4.2.1. Experimental Grouping, UC Model Establishment, and Drug Administration

After a 7-day acclimatization period, rats were randomly assigned into six groups (*n* = 8) using a computer-generated random number sequence: a control group; a model group; a positive control group (SASP, 100 mg/kg); and three RT2-treated groups at low (RT2_L_, 5 mg/kg), medium (RT2_M_, 10 mg/kg), and high doses (RT2_H_, 20 mg/kg). After a 24 h fast (with water provided ad libitum), rats were weighed and anesthetized. Colitis was induced in all groups, except for the control, by intrarectal administration of 0.04 mL/kg TNBS solution (100 mg/kg TNBS in 50% anhydrous ethanol). The solution was delivered approximately 8 cm into the anus via a glycerol-lubricated silicone tube [21]. Control rats received an equal volume of saline following an identical procedure. Except for those in the control group, all rats were administered 5% dextran sulfate sodium (DSS) for 24 h on day 10 to induce relapse. From day 1 to day 14, the treatment groups were administered SASP or RT2 aqueous solution daily via oral gavage. All drug administrations, sample collection, and data analyses were performed by investigators blinded to the group allocation. The blinding was maintained until the completion of all measurements.

#### 4.2.2. Body Weight, DAI, Colon Length, CDMI, and Spleen Coefficient

Body weight was recorded daily for all rats prior to gavage. The general behavioral status of the rat, including stool consistency and fecal bleeding, was observed daily. The DAI score, a widely used indicator of clinical severity in UC animal models, was assessed daily based on three parameters: body weight loss (0: none, 1: 1–5%, 2: 5–10%, 3: 10–15%, 4: >15%), stool consistency (0: normal, 2: loose stools, 4: diarrhea), and bleeding (0: negative, 2: mild bleeding, 4: severe bleeding). The DAI was calculated for each rat according to the following formula: DAI = (weight loss percentage score + stool consistency score + bleeding score)/3. Upon sacrifice, the colon was carefully excised and photographed, and its length was measured. The spleen was harvested and weighted, and the spleen coefficient was determined as spleen weight (mg) divided by body weight (g). Macroscopic colon injury was evaluated using the CMDI score, which assessed colonic adhesion; the number of ulcers; ulcer area; and the presence of intestinal wall congestion, edema, or thickening [22]. The detailed scoring criteria are presented in Table 2.

#### 4.2.3. Inflammatory and Oxidative Stress Factors

Colon tissue samples were homogenized in nine volumes of normal saline, and the homogenates were centrifuged at 13,000× *g* for 10 min to collect the supernatant. Levels of inflammatory cytokines (IL-1β, IL-6, IFN-γ, IL-10, IL-17A, and TNF-α) and oxidative stress markers (SOD, MDA, and GSH) in colon supernatants were quantified using commercial ELISA kits according to the manufacturer’s instructions.

#### 4.2.4. Histopathologic Examination

Segments of the distal colon were fixed in 10% neutral buffered formalin for 24 h, followed by dehydration through a graded ethanol series, paraffin embedding, and sectioning at 5 μm thickness. For H&E staining, sections were deparaffinized, rehydrated, stained with H&E, differentiated in acid alcohol, dehydrated, cleared in xylene, and mounted. For AB-PAS staining, deparaffinized and rehydrated sections were stained with Alcian Blue, oxidized in Periodic Acid, treated with Schiff’s reagent, and counterstained with hematoxylin. After differentiation and bluing in ammonia water, these sections were dehydrated, cleared, and mounted. All stained sections were examined and imaged using an optical microscope.

#### 4.2.5. Transmission Electron Microscopy Examination

Colon tissue samples were rinsed with PBS, opened longitudinally, and immediately fixed in 2.5% glutaraldehyde. Following primary fixation, the samples were post-fixed in 1% osmium tetroxide, dehydrated through a graded ethanol series, and embedded in epoxy resin. Ultrathin sections were then prepared, stained with uranyl acetate and lead citrate, and examined under a transmission electron microscope for ultrastructural analysis.

#### 4.2.6. Immunohistochemical Analysis of Tjs and Mucin

Immunohistochemistry was performed on murine colon sections prepared as described in Section 4.3.4. After deparaffinization, rehydration, and washing with PBS, sections were blocked with 10% goat serum and incubated overnight at 4 °C with primary antibodies against ZO-1, Claudin-1, Occludin, E-cadherin, and MUC2. Following incubation with appropriate HRP-conjugated secondary antibodies, antigen signals were visualized using DAB, and sections were counterstained with hematoxylin. The expression levels of the target proteins were evaluated by quantifying the average optical density (AOD) of positive staining using Image J software (version 1.54f).

#### 4.2.7. Flow Cytometric Analysis of Th17 and Treg Cells

T-cell subsets in mLNs were analyzed by flow cytometry. Briefly, mLNs were aseptically harvested, rinsed in PBS, and digested with collagenase type IV. The digestion was terminated by adding fetal bovine serum (FBS), and single-cell suspensions were obtained by gentle mechanical dissociation. Cells were stimulated with GolgiPlug™ for 4 h, counted, and adjusted to a density of 1 × 10^7^ cells/mL. For surface marker staining, cells were incubated with anti-rat FITC-conjugated CD4 and PE-conjugated CD25 antibodies at 4 °C for 30 min. After washing, cells were fixed and permeabilized using a commercial buffer. Intracellular staining was then performed with anti-rat Alexa Fluor 647-conjugated IL-17 and PerCP-conjugated Foxp3 antibodies at 4 °C for 40 min. Following a final wash, cells were resuspended in staining buffer, filtered through a cell strainer, and analyzed on a flow cytometer. Unstained and single-stained controls were included in each experiment for compensation and accurate gating.

#### 4.2.8. Tissue Distribution of RT2 in UC Rats

Following a 7-day acclimatization period, rats were randomly assigned into groups. A single model group (*n* = 6) and a treatment group (RT2_H_, 20 mg/kg) for pharmacokinetic assessment (*n* = 42) were established. Colitis was induced in all rats using the method described in Section 4.3.1. On day 14 of model establishment, rats in the treatment group received a single oral dose of RT2 via gavage. Subgroups of rats (*n* = 6 per time point) were then euthanized by cervical dislocation at 0.5, 1, 2, 4, 6, 10, and 24 h post-administration. Tissue samples, including the heart, liver, spleen, lung, kidney, stomach, small intestine, colon, muscle, fat, brain, uterus, ovary, testis, and epididymis, were promptly collected from each animal.

Approximately 50 mg of each tissue sample was accurately weighed and homogenized in normal saline. The homogenate was centrifuged at 12,000 rpm for 15 min at 4 °C. An equal volume of IS working solution was added to the resulting supernatant. The mixture was vortexed thoroughly and centrifuged again. The supernatant was collected and lyophilized, and the residue was reconstituted in 50 µL of methanol. Finally, all processed samples were analyzed using a validated LC-MS/MS method to determine the concentration of RT2.

### 4.3. Pharmacokinetic Studies of RT2

#### 4.3.1. Study Design and Sample Collection

Absorption study: To evaluate the absorption and systemic exposure of RT2, rats were assigned to four groups (*n* = 6 per group, with an equal number of males and females) after a 7-day acclimatization period: (1) RT2-ig-L (intragastric administration, 10 mg/kg), (2) RT2-ig-M (intragastric administration, 20 mg/kg), (3) RT2-ig-H (intragastric administration, 40 mg/kg), and (4) RT2-iv (intravenous administration, 4 mg/kg). Blood samples (~200 µL) were collected from the retro-orbital plexus into heparinized tubes at predetermined time points. For the intragastric groups, samples were collected pre-dose and at 0.25, 0.5, 1, 1.5, 2, 4, 6, 8, 12, 24, 48, 60, and 72 h post-dose. For the intravenous group, samples times were pre-dose and at 0.033, 0.083, 0.167, 0.333, 0.667, 1, 2, 4, 8, 12, 24, 48, and 60 h post-dose.

Distribution study: To investigate tissue distribution, a separate cohort of rats was used. Rats (equal numbers of males and females) were divided into 8 groups (*n* = 6 per group) by sampling time point (0.5, 1, 2, 4, 6, 10, and 24 h). A total of 42 rats received a single intragastric dose of RT2 (20 mg/kg), while the remaining 6 rats (control group) received an equivalent volume of saline. At each time point, rats were euthanized by cervical dislocation. Tissues including the heart, liver, spleen, lung, kidney, stomach, small intestine, colon, muscle, fat, brain, uterus, ovary, testis, and epididymis were immediately collected, rinsed with saline, blotted dry, and snap-frozen.

#### 4.3.2. Sample Preparation

Prior to LC-MS/MS analysis, all biological samples were processed via protein precipitation. The specific procedures for plasma and the tissue matrix are as follows.

Plasma samples: Whole blood was collected in heparinized tubes and centrifuged at 4000 rpm for 15 min at 4 °C to obtain plasma. A 100 µL aliquot of plasma was mixed with 400 µL of IS working solution (ginsenoside Re, 50 ng/mL in methanol) to precipitate proteins. After vortexing for 1 min, the mixture was centrifuged at 10,000 rpm for 15 min. The supernatant was collected, lyophilized, and reconstituted in 50 µL of methanol.

Tissue samples: Tissues were rinsed with saline and gently dried. Exactly 50 mg of tissue was homogenized in 450 µL of saline and centrifuged at 12,000 rpm for 15 min. An equal volume of IS working solution was added to the homogenate supernatant. The mixture was then vortexed and centrifuged, and the supernatant was processed in the same manner as the plasma samples (lyophilization and reconstitution in 50 µL of methanol).

#### 4.3.3. Preparation of Standard Solutions and Quality Control Samples

Based on a previous report that MR2 (an epimer of RT2) is metabolized in vivo via the removal of one or two sugar moieties [12], we hypothesized that RT2 undergoes similar deglycosylation to yield the metabolites RT5 and ocotillol, which were subsequently targeted for detection. The chemical structures of RT2, RT4, and ocotillol are displayed in the Figure S7 in Supplementary Materials. To quantify these compounds, standard stock solutions of RT2, RT5, and ocotillol were prepared in methanol at a concentration of 1.0 mg/mL. These stock solutions were then serially diluted with blank rat plasma and blank tissue homogenate to generate working standard solutions at concentrations of 5, 25, 50, 250, 500, 2500, and 5000 ng/mL. For the internal standard, a stock solution of ginsenoside Re was prepared in methanol (1.0 mg/mL) and subsequently diluted with methanol to obtain a working solution of 50 ng/mL.

QC samples were prepared in blank plasma and tissue homogenate at three concentration levels: low (LQC, 10 ng/mL), medium (MQC, 200 ng/mL), and high (HQC, 4000 ng/mL) by spiking appropriate volumes of the RT2, RT5, and ocotillol stock solutions. These QC samples were used to evaluate method performance during validation.

#### 4.3.4. Instrumentation, Chromatographic and Mass Spectrometric Conditions

Analysis was conducted using an AcquityTM UPLC system coupled with a XEVO TQ-S triple quadrupole tandem mass spectrometer equipped with an electrospray ionization (ESI) source (Waters Corporation, Milford, MA, USA).

Chromatographic conditions: Separation was achieved on an ACQUITY UPLC BEH C18 column (100 mm × 2.1 mm, 1.8 μm) (Waters Corporation, Milford, MA, USA) maintained at 31 °C. The mobile phase consisted of (A) water and (B) acetonitrile, delivered at a flow rate of 0.3 mL/min. The gradient elution program was as follows: 0–4.0 min, 20% → 100% B; 4.0–4.5 min, 100% B; 4.5–5.0 min, 100% → 20% B; 5.0–5.5 min, 20% B. The injection volume was 5 μL, and the autosampler temperature was set to 16 °C.

**Figure 8:**
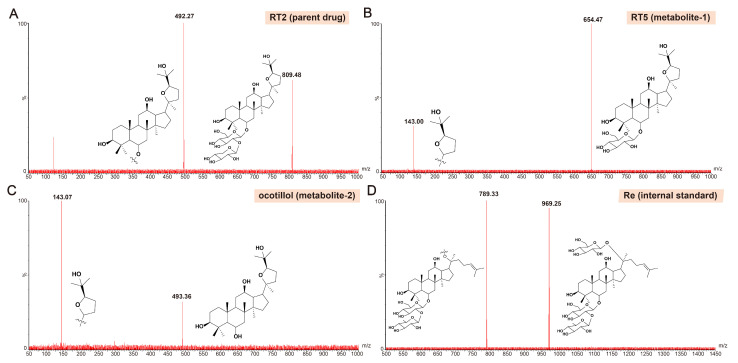
The product ion spectra for (**A**) RT2, (**B**) RT5, (**C**) ocotillol, and (**D**) Re.

Mass spectrometric conditions: RT2, its metabolites (RT5 and ocotillol), and the internal standard (ginsenoside Re) were detected in electrospray negative ionization (ESI^+^) mode using multiple reaction monitoring (MRM). The ion source parameters were as follows: capillary voltage, 3 kV; source temperature, 150 °C; desolvation temperature, 350 °C; desolvation gas flow, 650 L/h; cone gas flow, 150 L/h; nebulizer gas flow, 7 bar; and collision gas flow, 0.1 mL/min. The optimized MRM transitions, ion adducts, cone voltages, and collision energies for each analyte are summarized below: ① RT2: [M + Na]+, *m*/*z* 809.48 → 492.27 (cone voltage: 38 V; collision energy: 52 V); ② RT5: [M + H]+, *m*/*z* 654.47 → 143.00 (cone voltage: 24 V; collision energy: 16 V); ③ ocotillol: [M + H]+, *m*/*z* 493.36 → 143.07 (cone voltage: 24 V; collision energy: 16 V); ④ Re (IS): [M + Na]+, *m*/*z* 969.25 → 789.33 (cone voltage: 99 V; collision energy: 40 V). The product ion spectra for RT2, RT5, ocotillol, and Re are shown in Figure 8. Data acquisition and analysis were performed using Masslynx software (version 4.1).

#### 4.3.5. Method Validation

The bioanalytical method was validated according to the guidelines of the *Chinese Pharmacopoeia* (2025 Edition) [23].

Specificity: Specificity was evaluated by comparing chromatograms of the blank plasma/tissue homogenate; the blank matrix spiked with RT2, RT5, ocotillol, and the IS (ginsenoside Re) at the lower limit of quantification (LLOQ); and actual study samples (e.g., collected 2 h or 12 h post-dose). The method was considered specific if no significant interfering peaks were observed at the retention times of the analytes and the IS.

Calibration curve and linearity: Calibration curves for RT2, RT5, and ocotillol were established by plotting the peak area ratio of each analyte to the IS (y) against the nominal concentration (x, ng/mL). A weighted (1/x^2^) least squares linear regression model was applied. The linear range was defined from the lower limit of quantification (LLOQ) to the upper limit of quantification (ULOQ) with a correlation coefficient (r) exceeding 0.99.

LLOQ: The LLOQ was established as the lowest concentration on the calibration curve, set to adequately cover the pharmacokinetic profile (typically 3–5 half-lives or 1/10 to 1/20 of Cmax). At the LLOQ, the accuracy (relative error, RE) was required to be within 80%~120% of the nominal value, with a precision (relative standard deviation, RSD) not exceeding 20%.

Precision and accuracy: Intra-day and inter-day precision and accuracy were evaluated by analyzing six replicates of QC samples at low, medium, and high (LQC, MQC, and HQC) concentrations within a single day and over three consecutive validation days, respectively. The precision (RSD) for each level was required to be less than 15%, and accuracy (RE) was required to be within 85%~115% of the nominal values.

Dilution reliability: To confirm the reliability of sample dilution, blank plasma and tissue homogenate were spiked with RT2, RT5, and ocotillol at a concentration of 7500 ng/mL and then diluted 5-fold with the respective blank matrix to 1500 ng/mL. Six replicates of the diluted samples were analyzed. Accuracy was required to be within 85%~115%, with an RSD of <15%.

Extraction recovery and matrix effect: Extraction recovery and the matrix effect were evaluated at three QC concentration levels (LQC, MQC, and HQC) using six replicates each. The acceptable range for both was 85–115%. Extraction recovery was calculated by comparing the peak areas of analytes spiked into the blank matrix before extraction (A) with those spiked into the post-extraction supernatant (B). Recovery (%) = (B/A) × 100%. The matrix effect was assessed by comparing the peak area ratios of analytes to the IS in post-extraction spiked samples (B_analyte_/B_IS_) with those in pure methanolic solutions at equivalent concentrations (C_analyte_/C_IS_). Matrix factor (%) = [(B_analyte_/B_IS_)/(C_analyte_/C_IS_)] × 100%.

Stability: The stability of RT2, RT5, and ocotillol was investigated under various conditions using MQC samples. The conditions included: short term at room temperature (25 ± 2 °C for 24 h), long-term frozen (−80 °C for 1 month), after three freeze–thaw cycles (−80 °C to 25 °C), stock solution stability (4 °C for 1 month), and processed sample stability in an autosampler (4 °C for 72 h). Samples were considered stable if the mean concentration deviation from the nominal value was within ±15%.

Carryover: Carryover was assessed by injecting blank matrix samples immediately after three consecutive injections of ULOQ samples. The response in the blank at the retention times of the analytes was required to be ≤20% of the LLOQ response and ≤5% of the IS response.

### 4.4. Statistical Analysis

All data were analyzed using GraphPad Prism software (version 8.0). Results from pharmacodynamic studies are expressed as mean ± standard error of the mean (SEM), while pharmacokinetic data are expressed as mean ± standard deviation (SD). For comparisons among multiple groups, a one-way analysis of variance (ANOVA) was performed, followed by a two-tailed Student’s *t*-test for post hoc pairwise comparisons. A *p*-value of less than 0.05 was considered statistically significant.

## 5. Conclusions

This study provides a comprehensive pharmacodynamic and pharmacokinetic evaluation of RT2 in the context of UC. In a TNBS-induced rat model, RT2 treatment was associated with significant improvement of colitis symptoms, accompanied by restoration of intestinal mucosal barrier integrity and modulation of the Th17/Treg immune balance, as reflected by changes in relevant cytokine levels. Pharmacokinetically, RT2 exhibited rapid absorption, slow elimination, and preferential colonic distribution (with higher concentrations in the inflamed colon than in healthy rats). A biphasic plasma concentration–time profile was observed, which may be suggestive of enterohepatic recirculation; however, direct experimental confirmation is needed. Overall, this work transitions RT2 from a scarcely available natural product to a well-characterized lead compound by providing integrated pharmacodynamic and pharmacokinetic data. The observed association between colonic exposure and therapeutic efficacy supports the potential of RT2 as a gut-selective agent for UC, laying essential groundwork for future translational research and precision therapy.

## Figures and Tables

**Table 1:** Main pharmacokinetic parameters of RT2 in rat plasma after oral and intravenous administration (mean ± SD, *n* = 6).

Pharmacokinetic Parameters	RT2-ig-L (10 mg/kg)	RT2-ig-M (20 mg/kg)	RT2-ig-H (40 mg/kg)	RT2-iv (4 mg/kg)
AUC_0–t_ (μg·h/L)	3026.69 ± 209.92	6621.79 ± 570.89	12,724.14 ± 1335.64	8782.73 ± 436.16
AUC_0–∞_ (μg·h/L)	3207.37 ± 141.64	6929.95 ± 576.26	13,310.78 ± 1233.36	9647.54 ± 325.21
MRT_0–t_ (h)	15.30 ± 0.81	14.22 ± 0.80	14.84 ± 0.67	7.99 ± 0.52
MRT_0–∞_ (h)	18.96 ± 1.34	16.09 ± 0.58	16.37 ± 0.82	10.61 ± 2.22
t_1/2_ (h)	13.61 ± 1.60	12.69 ± 1.81	12.30 ± 1.02	16.94 ± 1.26
T_max_ (h)	2.00 ± 0.00	1.83 ± 0.26	1.92 ± 0.20	0.03 ± 0.00
V_d_ (L/kg)	61.51 ± 9.23	52.91 ± 7.10	53.79 ± 7.38	10.14 ± 0.76
CL (L/h/kg)	3.12 ± 0.14	2.90 ± 0.24	3.03 ± 0.28	0.41 ± 0.01
C_max_ (μg/L)	217.47 ± 19.29	427.57 ± 29.85	876.66 ± 59.77	4048.57 ± 347.14

**Table 2:** CMDI score criteria for colonic mucosal damage.

Score	Description of Mucosal Damage
0	No visible damage.
1	Focal, mild hyperemia and edema; mucosal surface intact, with no erosion or ulceration.
2	Diffuse hyperemia and edema with intestinal wall thickening; presence of mild erosion, but no ulceration.
3	Marked hyperemia and edema; moderate mucosal erosion; presence of a single ulcer.
4	Severe hyperemia and edema with surface necrosis; extensive mucosal erosion; multiple ulcers (largest ulcer < 1 cm in longitudinal diameter).
5	Features consistent with score 4, but with ulcers present in two or more distinct colonic regions (largest ulcer ≥ 1 cm).
6–10	Features consistent with score 5, with one or more of the following: ulcer longitudinal diameter > 2 cm, transmural necrosis, or multiple perforations. The score increases by 1 for each additional centimeter of major ulcer diameter beyond 2 cm or for the presence of severe complications.

## Data Availability

The original contributions presented in this study are included in the article/Supplementary Material. Further inquiries can be directed to the corresponding authors.

## Supplementary Materials

The following supporting information can be downloaded at: https://www.mdpi.com/article/10.3390/ph19040622/s1, Table S1. 1H NMR and 13C NMR (MeOD) chemical shifts of RT2; Table S2. Analytical method validation: Calibration curves, correlation coefficients, linear range, precision, and precision/accuracy at the LLOQ for RT2, RT5, and ocotillol across studied biological in matrix sample; Table S3. Precision and accuracy of RT2, RT5 and ocotillol in plasma, liver, and colon; Table S4. Dilution reliability of RT2, RT5 and ocotillol in plasma; Table S5. Extraction recovery and matrix effect of RT2, RT5 and ocotillol in plasma, liver, and colon tissue homogenates; Table S6. Stability of RT2, RT5 and ocotillol under various storage and handling conditions; Figure S1. HR-MS of RT2; Figure S2. 1H-NMR of RT2; Figure S3. 13C-NMR of RT2; Figure S4. HSQC of RT2; Figure S5. HMBC of RT2; Figure S6. Representative immunohistochemical staining of TJs (A,B) Claudin-1 and (C,D) E-cadherin. Data are presented as mean ± SEM. ^###^ *p* < 0.001 versus control group; * *p* < 0.05, ** *p* < 0.01 versus model group; ^Δ^ *p* > 0.05 versus SASP group; Figure S7. The chemical structures of RT2, RT4 and ocotillol; Figure S8. Presents representative LC-MS/MS chromatograms of RT2, its metabolites (RT5 and ocotillol), and the internal standard (Re, IS). (A) plasma, (B) liver and (C) colon samples.

## Abbreviations

The following abbreviations are used in this manuscript:

AB-PAS	Alcian Blue–Periodic Acid–Schiff
ADME	Absorption, distribution, metabolism, and excretion
CDMI	Chronic disease management index
DAI	Disease activity index
ELSD-HPLC	Evaporative light-scattering detection coupled with high-performance liquid chromatography
GSH-Px	Glutathione peroxidase
H&E	Hematoxylin and eosin
HMBC	Heteronuclear multiple bond correlation
HMQC	Heteronuclear multiple quantum coherence
HR-MS	High-resolution mass spectrometry
IFN-γ	Interferon-γ
IL-1β	Interleukin-1β
IS	Internal standard
LLOQ	Lower limit of quantification
MDA	Malondialdehyde
mLNs	Mesenteric lymph nodes
NMR	Nuclear magnetic resonance
PK	Pharmacokinetic
RT2	Pseudoginsenoside
QC	Quality control
SASP	Sulfasalazine
SPF	Specific pathogen-free
SOD	Superoxide dismutase
TEM	Transmission electron microscopy
TJs	Tight junction protein
TNF-α	Tumor necrosis factor-α
TNBS	2,4,6-trinitrobenzene sulfonic acid
UC	Ulcerative colitis
