# Supplementary material for: Development of Pseudoginsenoside RT2 as a Novel Gut-Selective Agent: Integrated Pharmacodynamic and Pharmacokinetic Evaluation of an Ocotillol Ginsenoside for Ulcerative Colitis
Source: Pharmaceuticals (Basel). 2026 Apr 15;19(4):622. doi: 10.3390/ph19040622 (PMC13119218; doi:10.3390/ph19040622)
Supplement: Supplementary file 1 [file pharmaceuticals-19-00622-s001.zip › pharmaceuticals-4235031-supplementary.pdf]

Supplementary material

Structural identification of RT2

A complete assignment of the carbon and proton signals of RT2 is provided in Supplementary Material **Table S1**.

Table S1. ^{1}H NMR and ^{13}C NMR (MeOD) chemical shifts of RT2.

C	NR1	C	NR1	H	NR1	H	NR1
1	40.27	6-O-Glc		1	1.84/1.08	6-O-Glc	
2	27.52	1'	103.82	2	1.59/1.54	1'	4.59
3	79.29	2'	80.02	3	3.63	2'	
4	40.49	3'	78.26	4	3.92	3'	3.47
5	62.01	4'	71.93	5	-	4'	3.52
6	79.69	5'	80.64	6	3.45	5'	4.12
7	45.16	6'	62.92	7	2.05/1.75	6'	3.84/3.65
8	41.90	Xyl		8	-	Xyl	
9	51.38	1''	103.88	9	1.58	1''	4.46
10	40.36	2''	75.57	10	-	2''	3.26
11	32.86	3''	77.64	11	1.90	3''	3.30
12	71.28	4''	72.35	12	3.28	4''	3.62
13	49.51	5''	66.93	13	1.67	5''	3.82/3.14
14	53.21			14	-		
15	31.32			15	1.72		
16	26.01			16	1.87		
17	49.74			17	1.74		
18	18.05			18	0.98		
19	17.29			19	0.99		
20	87.88			20	-		
21	27.04			21	1.28		
22	33.61			22	1.63		
23	29.66			23	2.21/2.02		
24	85.98			24	3.92		
25	71.74			25	-		
26	26.62			26	1.14		
27	26.71			27	1.23		
28	32.17			28	1.32		
29	16.45			29	1.10		
30	18.47			30	0.98		

Method validation of pharmacokinetic studies of RT2

Table S2. Analytical method validation: Calibration curves, correlation coefficients, linear range, precision, and precision/accuracy at the LLOQ for RT2, RT5, and ocotillol across studied biological in matrix sample.

Sample	Analyte	Calibration Curve	Correlation Coefficient (R^2)	Linear Range (ng/mL)	LLOQ (%)	
					Precision (RSD,%)	Accuracy (RE,%)
Plasma	RT2	y=0.0007x+0.0790	0.9981	5-5000	12.16	113.84
	RT5	y=0.0005x-0.0026	0.9956	5-5000	14.35	82.87
	ocotillol	y=0.0003x+0.0004	0.9963	5-5000	15.27	115.95
Heart	RT2	y=0.0008x+0.0605	0.9987	5-5000	11.29	85.24
	RT5	y=0.0005x+0.0012	0.9983	5-5000	17.12	119.36
	ocotillol	y=0.0003x-0.0005	0.9990	5-5000	12.79	118.21
Liver	RT2	y=0.0007x+0.0874	0.9987	5-5000	12.89	86.32
	RT5	y=0.0004x+0.0079	0.9990	5-5000	14.30	115.57
	ocotillol	y=0.0003x-0.0011	0.9997	5-5000	14.12	81.16
Spleen	RT2	y=0.0009x-0.0015	0.9996	5-5000	15.14	119.92
	RT5	y=0.0005x-0.0018	0.9961	5-5000	14.12	81.87
	ocotillol	y=0.0003x+0.0063	0.9973	5-5000	15.63	81.00
Lung	RT2	y=0.0009x-0.0028	0.9995	5-5000	14.11	86.16
	RT5	y=0.0005x+0.0042	0.9971	5-5000	12.78	84.67
	ocotillol	y=0.0002x-0.0003	0.9951	5-5000	12.85	115.75
Kidney	RT2	y=0.0007x+0.1003	0.9970	5-5000	16.49	80.06
	RT5	y=0.0005x+0.0004	0.9949	5-5000	13.42	84.30
	ocotillol	y=0.0002x+0.0131	0.9932	5-5000	12.47	82.97
Stomach	RT2	y=0.0008x+0.0828	0.9988	5-5000	16.08	119.86
	RT5	y=0.0005x-0.0009	0.9965	5-5000	12.74	118.65
	ocotillol	y=0.0003x-0.0046	0.9968	5-5000	15.47	119.26
Small intestine	RT2	y=0.0007x+0.0937	0.9946	5-5000	10.06	112.33
	RT5	y=0.0005x+0.0149	0.9983	5-5000	12.03	117.03
	ocotillol	y=0.0003x+0.0415	0.9946	5-5000	12.33	84.77
Colon	RT2	y=0.0007x+0.1024	0.9947	5-5000	11.56	85.81
	RT5	y=0.0005x+0.0165	0.9960	5-5000	12.09	82.26
	ocotillol	y=0.0003x+0.0023	0.9994	5-5000	12.39	114.35
Muscle	RT2	y=0.0008x-0.0013	0.9970	5-5000	11.99	86.59
	RT5	y=0.0005x-0.0013	0.9968	5-5000	16.33	119.58
	ocotillol	y=0.0003x+0.0267	0.9976	5-5000	17.67	119.84
Fat	RT2	y=0.0008x-0.0008	0.9978	5-5000	15.20	119.24
	RT5	y=0.0005x-0.0002	0.9952	5-5000	11.93	115.16
	ocotillol	y=0.0003x+0.0077	0.9975	5-5000	13.26	116.60
Brain	RT2	y=0.0007x+0.0945	0.9971	5-5000	15.44	81.78
	RT5	y=0.0004x+0.0262	0.9977	5-5000	15.94	81.85
	ocotillol	y=0.0003x-0.0023	0.9996	5-5000	12.02	114.73
Uterus	RT2	y=0.0008x+0.0635	0.9964	5-5000	12.42	85.67
	RT5	y=0.0005x+0.0079	0.9942	5-5000	11.83	117.72
	ocotillol	y=0.0003x-0.0009	0.9992	5-5000	13.17	114.45

Sample	Analyte	Calibration Curve	Correlation Coefficient (R^2)	Linear Range (ng/mL)	LLOQ (%)	
					Precision (RSD,%)	Accuracy (RE,%)
Ovary	RT2	y=0.0009x-0.0015	0.9987	5-5000	15.82	118.96
	RT5	y=0.0005x-0.0016	0.9961	5-5000	15.78	118.70
	ocotillol	y=0.0003x+0.0158	0.9911	5-5000	17.81	81.35
Testis	RT2	y=0.0009x-0.0031	0.9996	5-5000	11.68	114.14
	RT5	y=0.0005x+0.0285	0.9971	5-5000	11.47	114.07
	ocotillol	y=0.0003x-0.0005	0.9983	5-5000	12.64	81.01
Epididymis	RT2	y=0.0007x+0.0971	0.9970	5-5000	13.38	114.45
	RT5	y=0.0005x+0.0165	0.9978	5-5000	15.44	119.57
	ocotillol	y=0.0003x-0.0055	0.9979	5-5000	13.69	80.63

Table S3. Precision and accuracy of RT2, RT5 and ocotillol in plasma, liver, and colon.

Sample	Analyte	Con. (ng/mL)	Intra-Day			Inter-Day		
			Observed Con. (Mean ± SD, ng/mL)	Precision (RSD,%)	Accuracy (RE,%)	Observed Con. (Mean ± SD, ng/mL)	Precision (RSD,%)	Accuracy (RE,%)
Plasma	RT2	10	10.00 ± 1.31	13.06	85.37	10.36 ± 1.41	13.64	85.26
		200	205.55 ± 12.22	12.22	114.66	207.68 ± 25.26	12.16	86.34
		4000	4160.35 ± 432.47	10.39	113.10	4010.35 ± 406.44	10.13	112.92
	RT5	10	9.95 ± 1.40	14.08	85.62	9.96 ± 1.26	12.65	86.54
		200	202.00 ± 22.33	11.06	114.27	196.03 ± 20.38	10.40	112.79
		4000	3965.86 ± 429.89	10.84	111.82	3992.67 ± 396.61	9.93	86.75
	ocotillol	10	10.08 ± 1.38	13.69	86.62	10.04± 1.33	13.29	114.91
		200	194.81 ± 22.34	11.47	113.78	194.44 ± 20.90	10.75	87.74
		4000	3866.36 ± 415.21	10.74	87.92	3999.54 ± 202.88	5.07	94.66
Liver	RT2	10	10.01 ± 1.39	13.84	114.87	9.77 ± 1.27	13.00	114.48
		200	199.22 ± 23.63	11.86	86.52	197.01 ± 22.09	11.21	86.75
		4000	3955.25 ± 437.06	11.05	85.82	3962.55 ± 410.90	10.37	88.40
	RT5	10	10.05 ± 1.35	13.41	114.82	9.81 ± 1.33	13.56	85.28
		200	196.05 ± 20.46	10.44	112.40	199.33 ± 22.70	11.39	87.29
		4000	3967.36 ± 411.44	10.37	86.70	3868.35 ± 389.70	10.07	111.71
	ocotillol	10	9.98 ± 1.30	13.06	114.08	10.09 ± 1.32	13.10	85.94
		200	202.44 ± 22.05	10.89	112.34	193.38 ± 20.49	10.59	88.08
		4000	3943.91 ± 10.30	10.30	112.24	4001.77 ± 414.34	10.35	88.10
Colon	RT2	10	10.02 ± 1.35	13.51	114.34	10.00 ± 1.35	13.46	114.50
		200	197.32 ± 23.93	12.13	85.61	200.42 ± 23.99	11.97	86.02
		4000	4009.23 ± 454.98	11.35	112.81	3993.45 ± 460.80	11.54	87.08
	RT5	10	9.98 ± 1.31	13.12	114.18	9.93 ± 1.26	12.70	114.09
		200	196.42 ± 22.12	11.26	86.08	198.30 ± 10.13	10.12	88.51
		4000	4067.39 ± 419.16	10.31	112.72	3919.96 ± 212.10	9.87	113.21
	ocotillol	10	9.63 ± 1.28	13.25	86.35	10.34 ± 1.32	12.76	114.30
		200	205.65 ± 10.80	10.80	87.38	200.38 ± 20.37	10.17	111.03
		4000	3926.26 ± 359.70	9.16	110.01	3882.16 ± 376.86	9.71	110.49

Table S4. Dilution reliability of RT2, RT5 and ocotillol in plasma.

Analyte	Mean ± SD (ng/mL)	Precision（RSD, %）	Accuracy（RE, %）
RT2	1482.32 ± 63.02	10.61	88.12
RT5	1511.41 ± 93.02	10.10	87.52
ocotillol	1490.40 ± 108.62	10.35	112.49

Table S5. Extraction recovery and matrix effect of RT2, RT5 and ocotillol in plasma, liver, and colon tissue homogenates.

Sample	Analyte	Nominal Concentration (ng/mL)	Recovery Effect (%)		Matrix Effect (%)	
			Mean ± SD	RSD	Mean ± SD	RSD
Plasma	RT2	10	94.19 ± 10.42	11.06	93.52 ± 10.41	11.13
		200	96.78 ± 10.51	10.86	95.09 ± 10.00	10.51
		4000	98.11 ± 9.34	9.52	96.67 ± 9.03	9.34
	RT5	10	93.91 ± 12.10	12.89	95.08 ± 12.80	13.46
		200	94.85 ± 10.22	10.78	100.59 ± 11.45	11.38
		4000	97.65 ± 9.14	9.36	97.16 ± 9.56	9.83
	ocotillol	10	93.91 ± 12.10	12.89	95.08 ± 12.80	13.46
		200	94.85 ± 10.22	10.78	100.59 ± 11.45	11.38
		4000	97.65 ± 9.14	9.36	97.16 ± 9.56	9.84
Liver	RT2	10	97.28 ± 11.87	12.20	95.73 ± 11.17	11.67
		200	95.25 ± 10.00	10.50	97.26 ± 9.38	9.64
		4000	97.38 ± 9.79	10.05	100.91 ± 8.85	8.77
	RT5	10	93.50 ± 10.14	10.84	97.73 ± 10.82	11.07
		200	94.58 ± 9.63	10.18	97.64 ± 10.05	10.29
		4000	93.39 ± 7.85	8.41	95.85 ± 8.90	9.29
	ocotillol	10	94.05 ± 12.86	13.68	94.32 ± 11.96	12.68
		200	94.25 ± 9.59	10.18	97.68 ± 10.47	10.72
		4000	98.23 ± 9.06	9.22	96.86 ± 8.81	9.10
Colon	RT2	10	94.08 ± 12.05	12.81	102.88 ± 12.24	11.90
		200	97.92 ± 11.48	11.75	97.22 ± 9.78	10.06
		4000	96.29 ± 10.06	10.45	95.95 ± 8.67	9.03
	RT5	10	93.86 ± 10.77	11.48	94.33 ± 11.69	12.40
		200	94.20 ± 9.50	10.09	100.66 ± 11.94	11.87
		4000	95.35 ± 9.13	9.58	95.70 ± 9.36	9.78
	ocotillol	10	93.48 ± 11.32	12.12	93.41 ± 11.28	12.07
		200	95.72 ± 10.32	10.78	98.65 ± 10.32	10.46
		4000	97.24 ± 10.04	10.33	98.91 ± 9.53	9.63

Table S6. Stability of RT2, RT5 and ocotillol under various storage and handling conditions.

Samples	Condition	Analyte	LQC		HQC	
			**Precision (RSD, %)**	**Accuracy (RE, %)**	**Precision (RSD, %)**	**Accuracy (RE, %)**
Plasma	Short-term stability (25±2 °C, 24 h)	RT2	10.28	85.36	8.63	87.62
		RT5	12.54	86.52	10.13	112.65
		ocotillol	12.64	87.03	10.35	89.87
	Long-term stability (−80 °C, 1 month)	RT2	10.72	112.72	9.70	86.10
		RT5	12.06	85.53	11.98	113.62
		ocotillol	11.01	114.25	10.39	88.19
	Three freeze-thaw cycles	RT2	10.87	85.93	10.05	87.78
		RT5	13.14	85.89	12.17	87.16
		ocotillol	12.76	85.72	11.02	88.03
	Stock solution (4°C, 1 month)	RT2	10.25	85.30	9.67	90.87
		RT5	11.20	85.17	9.50	105.96
		ocotillol	10.14	113.99	9.79	89.44
	Auto-sampler stability (4 °C, 12 h)	RT2	9.76	113.06	9.00	90.34
		RT5	10.05	86.25	8.62	113.62
		ocotillol	10.26	87.01	9.94	111.42
Liver	Short-term stability (25±2 °C, 24 h)	RT2	12.85	114.62	10.45	110.57
		RT5	13.68	89.52	9.18	111.69
		ocotillol	12.29	88.50	10.16	90.15
	Long-term stability (−80 °C, 1 month)	RT2	10.51	110.99	10.23	87.38
		RT5	12.67	86.95	10.04	89.05
		ocotillol	10.93	110.84	10.81	112.10
	Three freeze-thaw cycles	RT2	13.51	114.52	11.48	113.87
		RT5	14.12	85.61	10.86	88.19
		ocotillol	13.16	85.77	11.21	114.42
	Stock solution (4°C, 1 month)	RT2	11.00	86.02	9.60	110.42
		RT5	10.25	85.07	9.03	111.36
		ocotillol	12.32	86.56	9.99	91.32
	Auto-sampler stability (4 °C, 12 h)	RT2	10.43	110.43	9.23	89.07
		RT5	10.69	85.98	10.18	89.71
		ocotillol	11.40	87.48	9.14	114.63
Colon	Short-term stability (25±2 °C, 24 h)	RT2	12.48	88.49	10.03	86.12
		RT5	12.04	85.74	9.62	114.99
		ocotillol	12.22	113.83	9.26	88.64
	Long-term stability (−80 °C, 1 month)	RT2	11.92	111.59	11.24	86.59
		RT5	11.47	87.83	9.76	86.64
		ocotillol	10.39	114.14	9.97	85.58
	Three freeze-thaw cycles	RT2	12.86	87.03	12.82	114.77
		RT5	13.72	88.71	10.83	109.38
		ocotillol	12.53	85.39	10.92	87.61
	Stock solution (4°C, 1 month)	RT2	10.95	114.59	9.94	112.77
		RT5	10.16	110.72	9.54	86.35
		ocotillol	10.14	85.57	10.29	88.53

Samples	Condition	Analyte	LQC		HQC	
			Precision (RSD, %)	Accuracy (RE, %)	Precision (RSD, %)	Accuracy (RE, %)
	Auto-sampler stability (4 ℃, 12 h)	RT2	10.53	112.21	9.34	89.44
		RT5	9.03	110.61	8.39	113.48
		ocotillol	10.75	111.66	9.21	107.65

Structural identification of RT2

The chemical structure of RT2 was unequivocally confirmed through a comprehensive spectroscopic analysis, which included HR-MS, along with ^{1}H-NMR, ^{13}C-NMR, HMQC, and HMBC experiments.

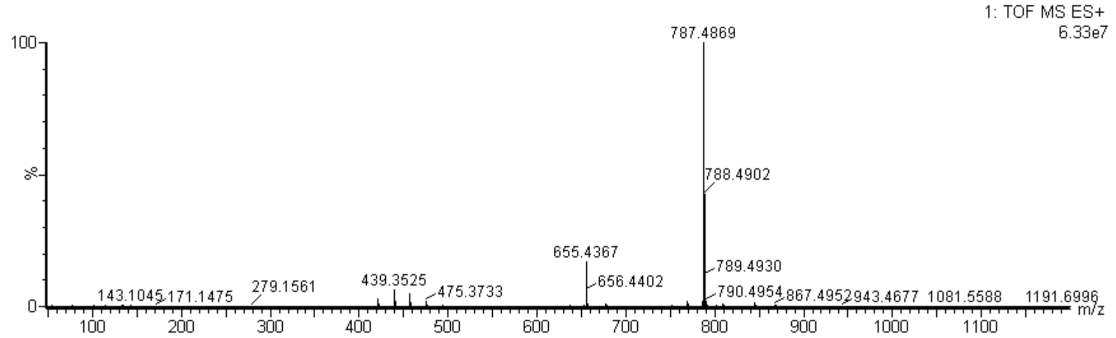

Figure S1. HR-MS of RT2.

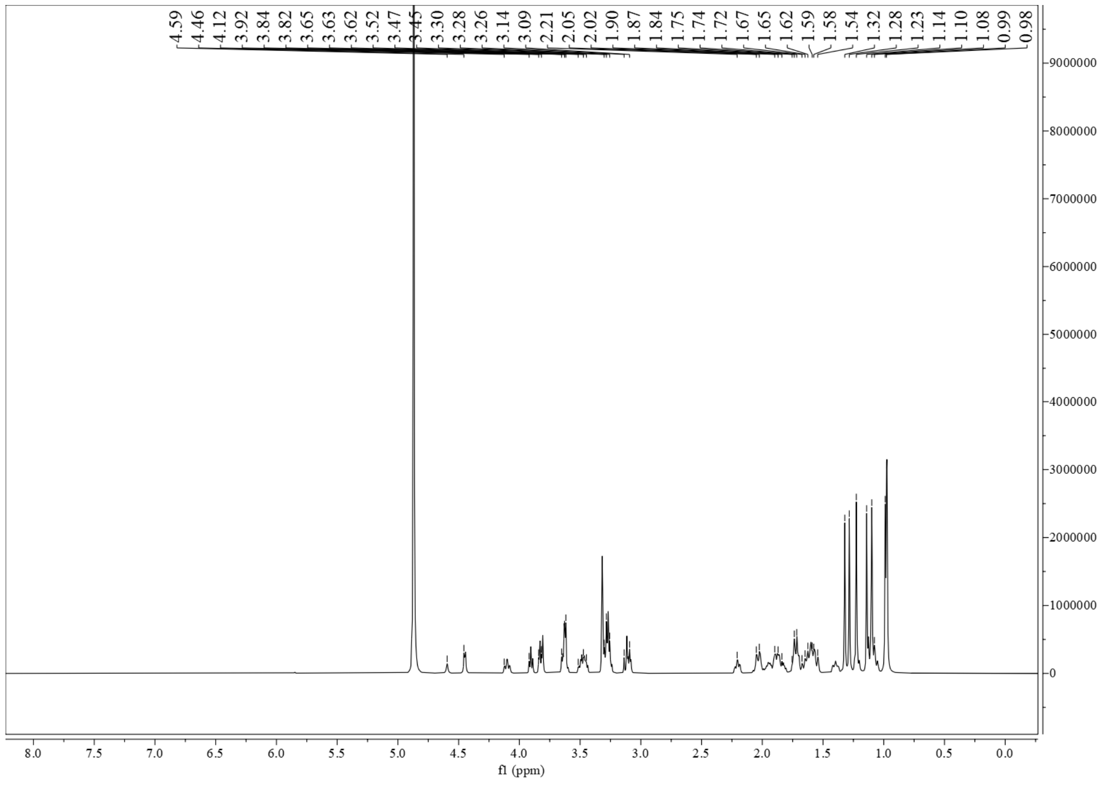

Figure S2. ^{1}H-NMR of RT2.

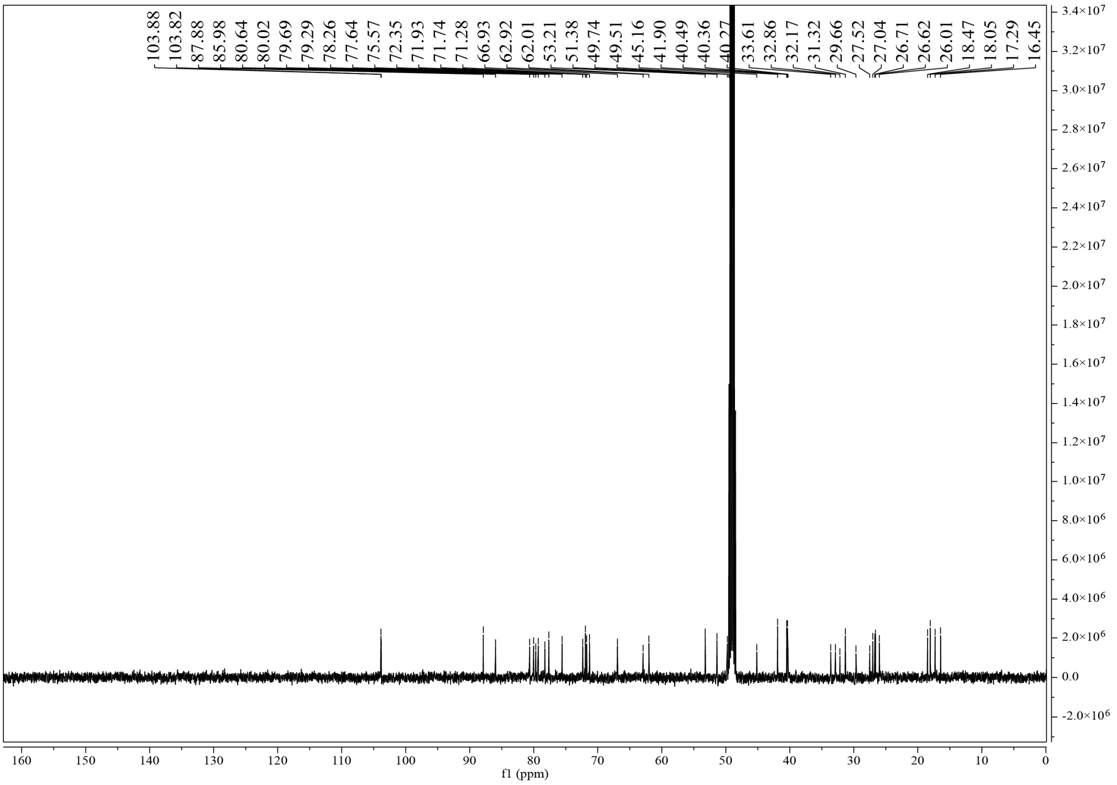

Figure S3. ^{13}C-NMR of RT2.

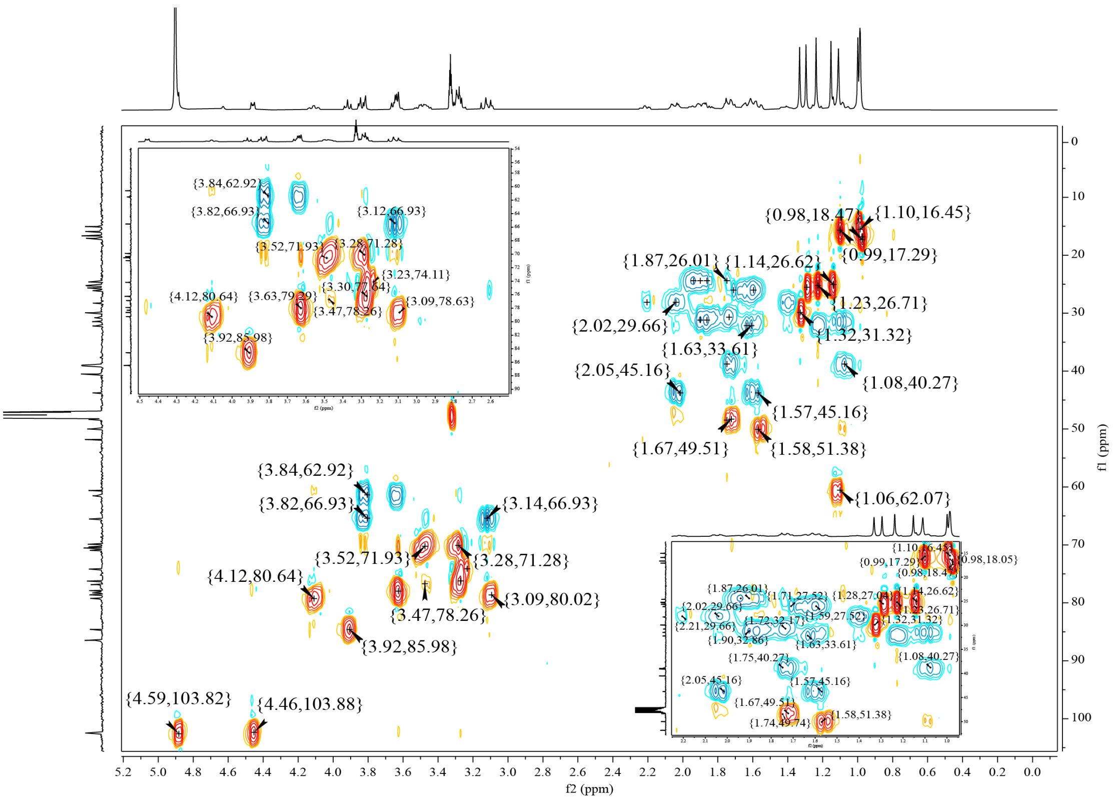

Figure S4. HSQC of RT2.

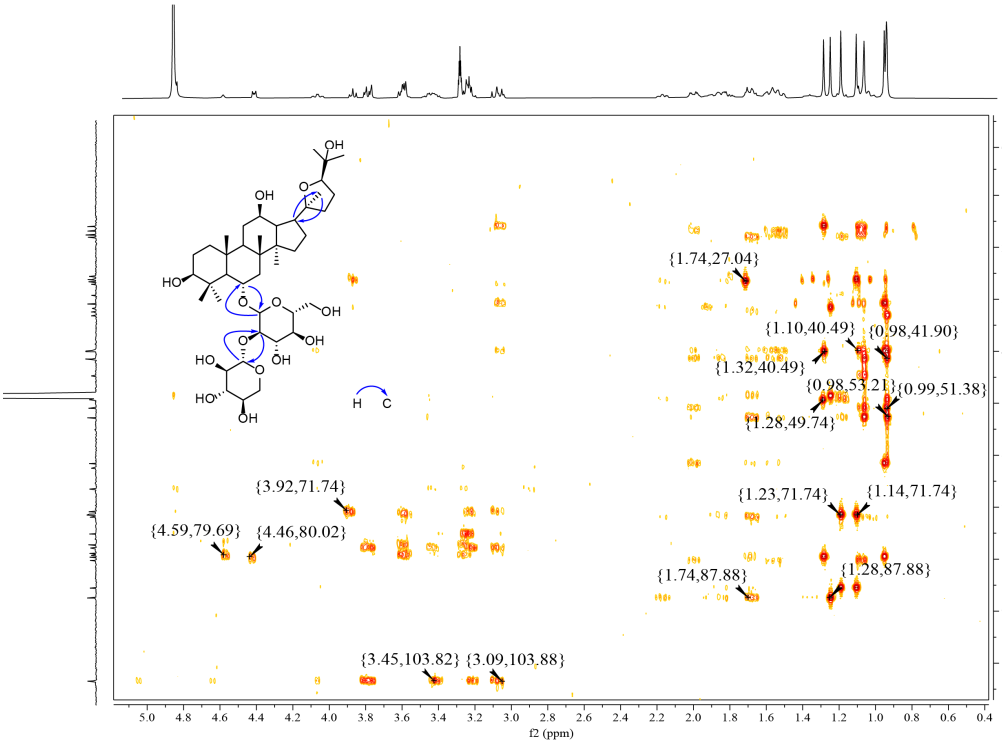

Figure S5. HMBC of RT2.

Immunohistochemical analysis of Tjs

Immunohistochemical staining was performed to evaluate the expression and localization of key TJs (Claudin-1 and E-cadherin). As shown in **Figure S6**, immunohistochemical staining for TJs control samples exhibited strong and continuous immunoreactivity for all markers, indicating intact mucosal integrity. In stark contrast, the model group showed markedly attenuated and discontinuous staining patterns, consistent with severe impairment of the intestinal barrier. Treatment with either SASP or RT2 effectively restored the intensity and continuity of the staining for these proteins. Quantitative analysis further confirmed these observations.

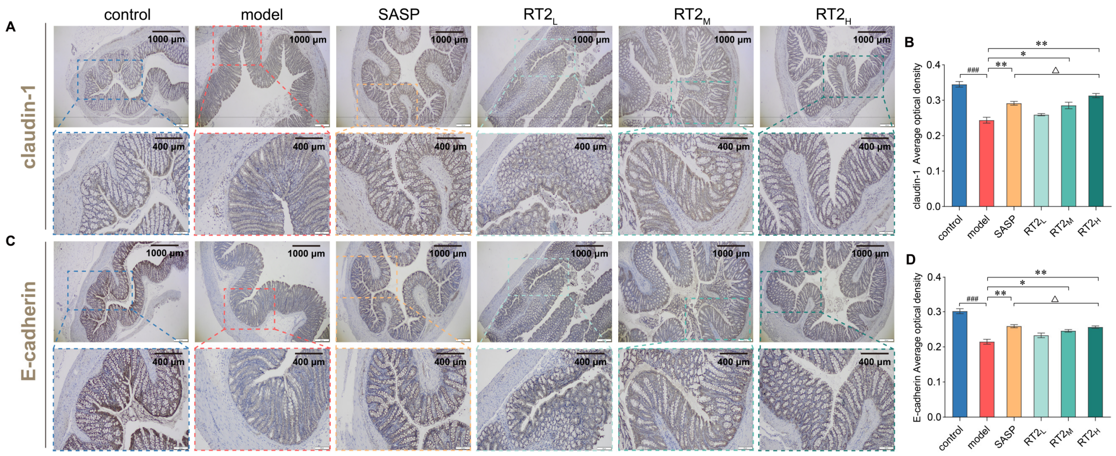

Figure S6. Representative immunohistochemical staining of TJs (A-B) Claudin-1 and (C-D) E-cadherin. Data are presented as mean ± SEM. ###p < 0.001 versus control group; *p < 0.05, **p < 0.01 versus model group; △p > 0.05 versus SASP group.

Preparation of standard solutions and quality control samples

The chemical structures of RT2, RT4 and ocotillol were in the supplementary materials **Figure S7**.

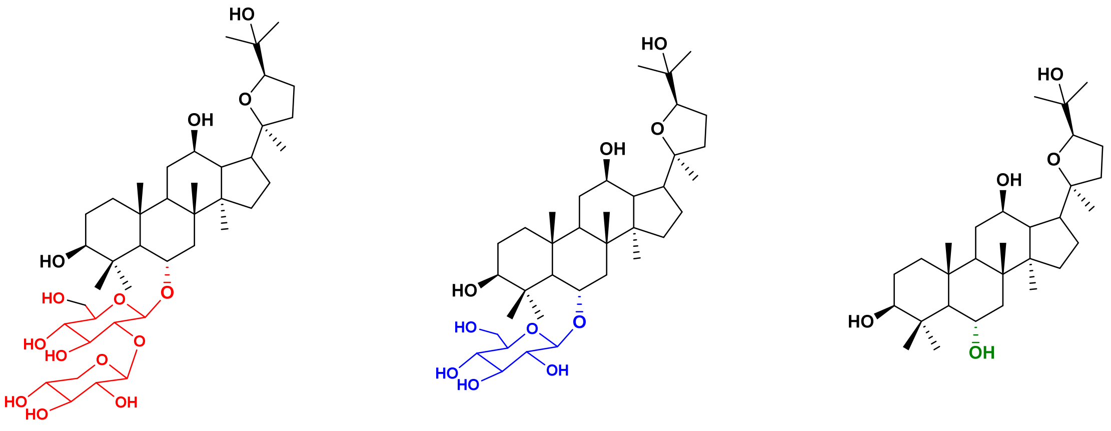

pseudoginsenoside RT2 pseudoginsenoside RT5 ocotillol

Figure S7. The chemical structures of RT2, RT4 and ocotillol.

Validation of the liquid chromatography-tandem mass spectrometry (LC-MS/MS) analytical method

Under the optimized chromatographic conditions, RT2, RT5, ocotillol, and the internal standard (IS) (Re) exhibited sharp peaks with retention times of 2.14, 2.22,

3.21, and 1.58 min, respectively. Representative chromatograms of blank matrices (plasma, liver and colon), blank matrices spiked with the analytes and IS, and actual study samples collected at 2 h or 4-8 h post-oral administration of RT2 were shown in **Figure S8**. No significant endogenous interference was observed at the retention times corresponding to the analytes or the IS in any of the tested matrices. The baseline remained stable across all chromatograms. These results confirm the high specificity of the developed method for the reliable quantification of RT2 and its metabolites in complex biological samples.

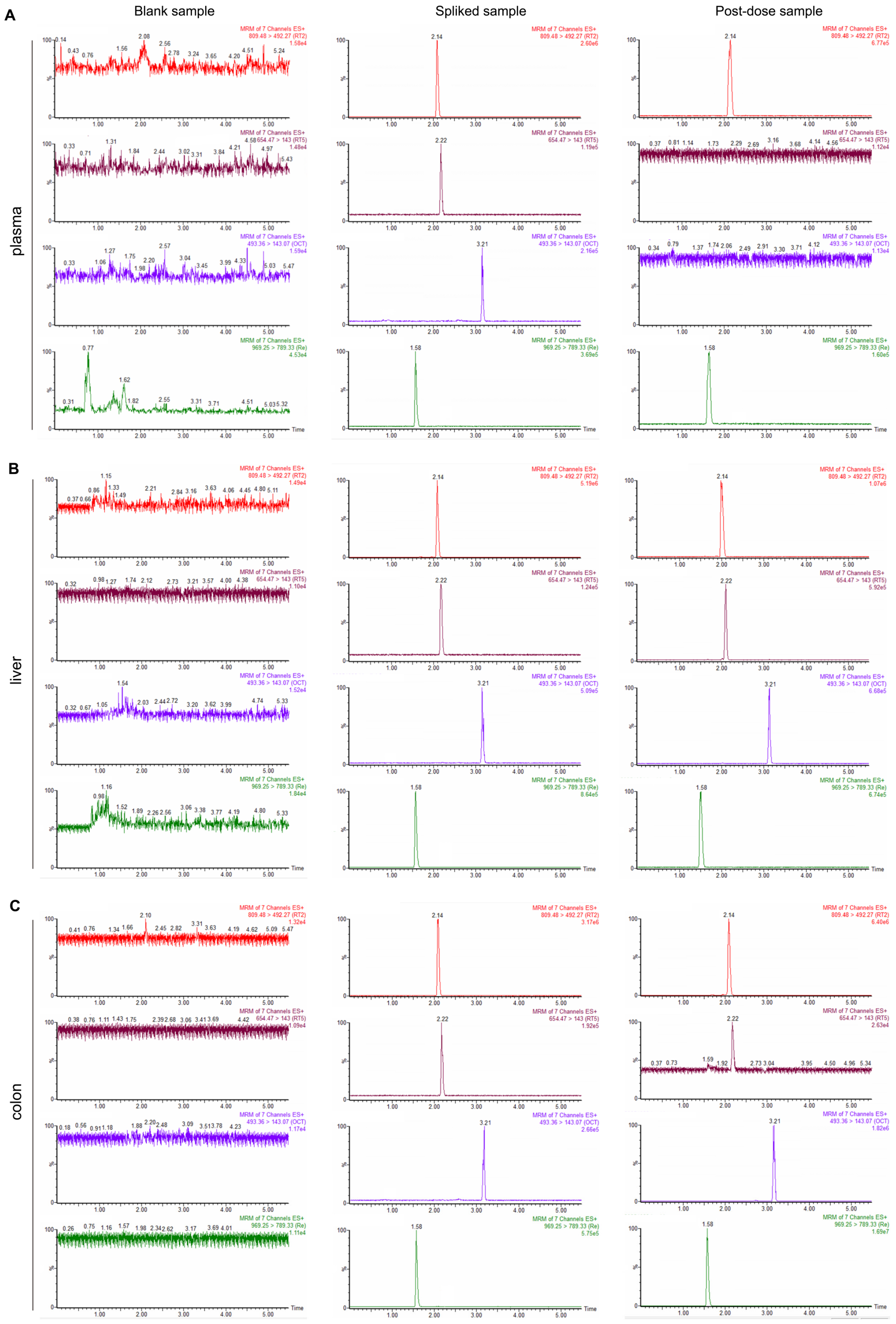

Figure S8. Presents representative LC-MS/MS chromatograms of RT2, its metabolites (RT5 and ocotillol), and the internal standard (Re, IS). (A) plasma, (B) liver and (C) colon samples.
